# Host-Associated Genetic Differentiation in the Face of Ongoing Gene Flow: Ecological Speciation in a Pathogenic Parasite of Freshwater Fish

**DOI:** 10.1093/molbev/msaf163

**Published:** 2025-07-08

**Authors:** Masoud Nazarizadeh, Milena Nováková, Jakub Vlček, Jan Štefka

**Affiliations:** Faculty of Science, University of South Bohemia in České Budějovice, České Budějovice 37005, Czechia; Institute of Parasitology, Biology Centre, CAS, České Budějovice 37005, Czechia; Institute of Parasitology, Biology Centre, CAS, České Budějovice 37005, Czechia; Faculty of Science, University of South Bohemia in České Budějovice, České Budějovice 37005, Czechia; Institute of Parasitology, Biology Centre, CAS, České Budějovice 37005, Czechia; Faculty of Science, University of South Bohemia in České Budějovice, České Budějovice 37005, Czechia; Institute of Parasitology, Biology Centre, CAS, České Budějovice 37005, Czechia

**Keywords:** host-associated genetic differentiation, gene flow, ecological speciation, differential gene expression, sympatric evolution

## Abstract

Adaptive evolution in response to varying environments, leading to population divergence, is among the most intriguing processes of speciation. However, the extent to which these adaptive processes effectively drive population divergence amidst ongoing gene flow remains controversial. Our study addresses this by analyzing population genetic structure, gene flow, and genomic divergence between lineages of a tapeworm parasite (Ligula intestinalis) isolated from sympatric fish hosts. This parasite, which must overcome host immunological defenses for successful infection, significantly impacts host health. Utilizing genome-wide Single Nucleotide Polymorphisms (SNPs) and transcriptome data, we investigated whether host species impose distinct selection pressures on parasite populations. Genetic clustering analyses revealed clear divergence, with parasites from bream (*Abramis brama*) forming a distinct genetic cluster separate from those infecting roach (*Rutilus rutilus*), rudd (*Scardinius erythrophthalmus*), and bleak (*Alburnus alburnus*). Demographic modeling indicated isolation with continuous gene flow as the most plausible scenario for this divergence. Selection analyses identified 896 SNPs under selection, displaying low to moderate nucleotide diversity and genetic divergence compared with neutral loci. Transcriptome profiling supported these findings, revealing distinct gene expression profiles between parasite populations. Examination of selected SNPs and differentially expressed genes identified candidate genes linked to immune evasion mechanisms, potentially driving ecological speciation. This research highlights the interplay of host specificity, population demography, and disruptive selection in ecological speciation. By dissecting genomic factors, our study improves the understanding of mechanisms facilitating population divergence despite ongoing gene flow.

## Introduction

The study of ecological speciation facilitates a comprehensive understanding of the complex interplay between evolutionary mechanisms and ecological interactions (Rundle and Nosil 2005; Feder et al. 2012; Nosil 2012). This form of speciation occurs when divergent selection pressures, associated with environmental factors, induce reproductive isolation (Nosil 2012). In allopatric situations, reproductive isolation may arise due to the accumulation of genetic variation, independently of selection pressures and adaptation to local conditions (Schluter 1996; Howard and Berlocher 1998; Orr and Smith 1998). Conversely, in sympatric scenarios where populations occupy the same geographic area, reproductive isolation typically stems from reinforcement processes dampening gene flow between emerging population lineages (Bush 1994; Pfennig 2016; Anderson et al. 2023).

Despite growing research in ecological speciation, our understanding of this process in parasites with complex life cycles remains limited (Poulin 2011; Brunner and Eizaguirre 2016; Henrich and Kalbe 2016). These parasites present unique research opportunities due to their multihost life cycles, which expose them to a variety of ecological pressures (Combes 2001; Poulin 2011). The complexity in their life cycles arises as these parasites must adapt to different hosts, each presenting a distinct set of ecological challenges, such as varied immune responses, habitats, and behaviors (Auld and Tinsley 2015). Interestingly, the unique ecological conditions of parasites, such as their strict habitat selection and potential for intra-host speciation, might make sympatric speciation a more common mode of speciation for parasites compared with free-living animals (McCoy 2003; Le Gac and Giraud 2004). Previous studies suggested that speciation by disruptive selection for host choice could play a crucial role in sympatric speciation in parasites (de Meeûs et al. 1995; Duffy et al. 2008; Fournier and Giraud 2008; Hébert et al. 2016; Santacruz et al. 2020). This selection is often driven by ecological differences among hosts, such as immune defenses, behavior, or physiology, that impose divergent pressures on parasite populations. For example, *Columbicola* lice diverge by host body size and grooming (Malenke et al. 2009), while *Vidua* brood parasitic indigobirds speciated by imprinting on host-specific song and chick traits (Sorenson et al. 2003). Experimental evolution showed feather lice to adapt their color to match white or black pigeon hosts under divergent selection from plumage color and host's preening (Baldwin-Brown et al. 2025). Even host diet can shape parasite traits, as seen in microbiome differences in *Philornis downsi* larvae across finch species (Ben-Yosef et al. 2017).

Over time, host-related selective pressure could result in the emergence of specialized subpopulations of parasites, each being adapted to a distinct host. Eventually, these subpopulations may become reproductively isolated, leading to sympatric speciation (McCoy 2003). Furthermore, parasites may exhibit hard selective sweeps in response to various selective pressures, such as host immune response after a host-switch, drug treatments, or competition among parasites for host resources (Ebert and Fields 2020; Le Pennec et al. 2024). When a beneficial mutation arises that enhances the parasite's ability to cope with one of these pressures, that allele might rapidly become predominant in the population. This rapid adaptation could lead to the formation of a specialized subpopulation of parasites, attuned to a specific host or set of conditions (Ebert and Fields 2020). Despite advances in genomic tools, allowing detailed studies of ecological speciation in many free living organisms (e.g. Riesch et al. 2017; Marques et al. 2019a; Kautt et al. 2020; Tavares et al. 2022) a gap persists in our understanding of the interplay of ecological speciation mechanisms in the context of parasites, particularly those possessing complex life cycles, including the description of precise genomic factors behind the diversification.

Our species of choice to study the ecological and genomic mechanisms at play, *Ligula intestinalis* (Cestoda), is a widespread diphyllobothriidean tapeworm and a valuable model for understanding the complex dynamics of parasitic speciation (Bouzid et al. 2008a; Štefka et al. 2009; Hoole et al. 2010; Nazarizadeh et al. 2022, 2023). It possesses a complex life cycle, beginning with the hatching of eggs into coracidia larvae in freshwater. These larvae then infect copepods and transform into procercoid larvae. Subsequently, after ingestion of the infected copepod by the second intermediate host, the planktivorous fish, the larvae gain body mass and develop into a plerocercoid stage inside the fish's body cavity. The life cycle culminates in a piscivorous bird, the final host, where the tapeworm reaches maturity and reproduces (Dubinina 1980). While the adult phase lasts only a few days, plerocercoid phase, involving the second intermediate host, is of particular interest because this is where the parasite spends most of its life time and encounters a range of ecological variables and host-specific selective pressures, such as differing immune responses and ecological habitats provided by various species of freshwater fish (Dubinina 1980; Williams and Hoole 1995; Halimi et al. 2013). Given that distinct parasite lineages are specialized to particular intermediate hosts, multiple genetically differentiated strains or closely related species have been identified within the same water bodies (Olson et al. 2002; Štefka et al. 2009; Nazarizadeh et al. 2023). For instance, Olson et al. (2002) identified significant genetic variations in *Ligula* populations between two sympatric fish hosts in Lough Neagh, Northern Ireland. Furthermore, recent phylogeographic research has revealed that this parasite has undergone multiple modes of speciation, including both sympatric and allopatric, resulting in its diversification into at least ten distinct evolutionary lineages across various biogeographical realms (see supplementary fig. S1, Supplementary Material online and Fig. 2 in Nazarizadeh et al. 2023). This ecological complexity and diversity in host use create an ideal setting for examining how speciation occurs in response to ecological factors, particularly given the parasite's capacity to adapt to different intermediate host species.

Of all ten known evolutionary lineages, Lineage A is particularly noteworthy due to its wide distribution in western Palearctic and wide fish host spectrum, including cyprinids such as freshwater bream (*Abramis brama*), white bream (*Blicca bjoerkna*), roach (*Rutilus rutilus*), rudd (*Scardinius erythrophthalmus*), bleak (*Alburnus alburnus*), minnow *(Phoxinus phoxinus*), chub (*Squalius cephalus*), and crucian carp (*Carassius carassius*) (Bouzid et al. 2008a, 2008b; Štefka et al. 2009; Nazarizadeh et al. 2023). Interestingly, it has been documented that host preferences vary considerably in different water bodies (Loot et al. 2002; Zhokhov and Pugacheva 2012; Nazarizadeh et al. 2022). For instance, a study in south-western France observed that *L. intestinalis* predominantly infects roach populations, even in the presence of other potential hosts (Loot et al. 2002). This variation in host specificity may indicate the development of host-specific races, suggesting adaptive differentiation within Lineage A. Given its ecological diversity and the observed patterns of host specificity, Lineage A provides a valuable model for investigating the mechanisms of divergent selection pressures and ecological isolation, contributing to our understanding of parasite evolution and speciation.

In our study, we aim to examine divergent selection pressures and ecological isolation mechanisms that might influence parasite adaptations and diversity in the presence of gene flow, using *L. intestinalis* Lineage A populations as an example. Utilizing a comprehensive approach that involves sampling in the areas where all hosts coexist in sympatry, followed by the analysis of genome-wide Single Nucleotide Polymorphisms (SNPs) and transcriptome data, we strive to: (i) ascertain whether host specificity in Lineage A can potentially influence the population structure of parasites in the absence of geographical separation, and (ii) identify potential genomic signatures indicative of host specialization within identified *Ligula* populations through selection analyses. Finally, we aim (iii) characterize host-associated adaptive divergence at the gene-expression level by establishing a comprehensive reference transcriptome and quantifying differential gene expression (DGE) between parasites infecting different sympatric hosts.

## Results

### Genomic Diversity

**Fig. 1.**
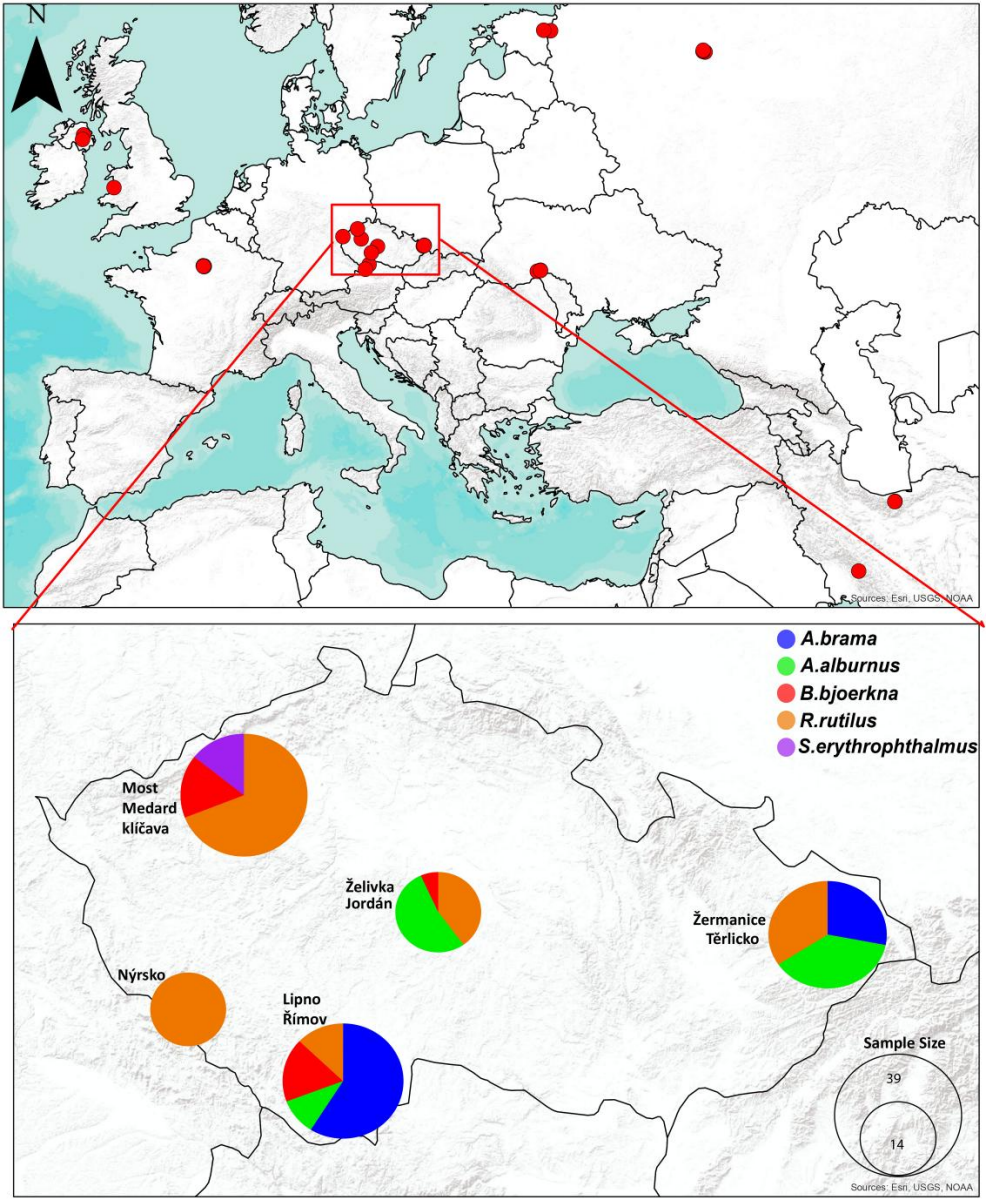
Sampling locations of the *L. intestinalis* Lineage A across the Palearctic (from Nazarizadeh et al. 2023, also analysed here). Inset: Sampling localities in Czechia, color-coded to reflect the five most prevalent host species. Sampling sites in proximity to each were amalgamated into a single circle. The size of each circle correlates with the sample size.

We analyzed 145 samples of *L. intestinalis* from eight fish host species across the distribution of Lineage A using genome-wide SNPs (see supplementary table S1, Supplementary Material online and Fig. 1). Using Dataset A (which contains all samples), we then compared the genetic characteristics of these parasite populations across different fish host species within *L. intestinalis* Lineage A (Table 1). Observed heterozygosity (HO) ranged from 0.093 in *S. orientalis* to 0.107 in *R. rutilus*, although overall, HO values were relatively similar across most hosts. Expected heterozygosity (HE) was highest in *R. rutilus* (0.131) and lowest in *S. orientalis* (0.109), again showing a relatively narrow range. Watterson's theta (θ_W_) varied more broadly, from 0.123 in *S. orientalis* up to 0.174 in *R. rutilus*, suggesting a moderately higher number of segregating sites in the parasite populations infecting *R. rutilus*.

**Table 1:** Genetic diversity of *L. intestinalis* Lineage A parasite populations across different host species

	Hosts	Locality	*N*	HO	HE	sMLH	θ_W_	Fis
*L. intestinalis* Lineage A	*R. rutilus*	Czechia, France, Ireland, Ukraine	73	0.107 (0.005)	0.131	1.028 (0.046)	0.174	0.100 (0.040)
	*A. brama*	Czechia, Estonia, Russia,	27	0.097 (0.013)	0.120	0.9370 (0.132)	0.140	0.0962 (0.127)
	*A alburnus*	Ukraine, Czechia	21	0.106 (0.007)	0.126	1.015 (0.074)	0.149	0.085 (0.066)
	*B. bjoerkna*	Czechia	12	0.102 (0.005)	0.119	0.976 (0.051)	0.134	0.054 (0.049)
	*S. erythrophthalmus*	Czechia	5	0.106 (0.002)	0.116	1.00 (0.022)	0.139	−0.008 (0.022)
	*S. orientalis*	Iran	5	0.093 (0.010)	0.109	0.891 (0.096)	0.123	0.060 (0.098)
	*C. carassius*	Ukraine	1	–	–	–	–	–
	*P. Phoxinus*	United Kingdom	1	–	–	–	–	–

Abbreviations: – not analysed; Fis, Inbreeding coefficient; HE, expected heterozygosity; HO, Observed heterozygosity; sMHL, standardized multi locus heterozygosity.

Numbers in parentheses represent standard deviations.

### Population Genetic Structure

**Fig. 2.**
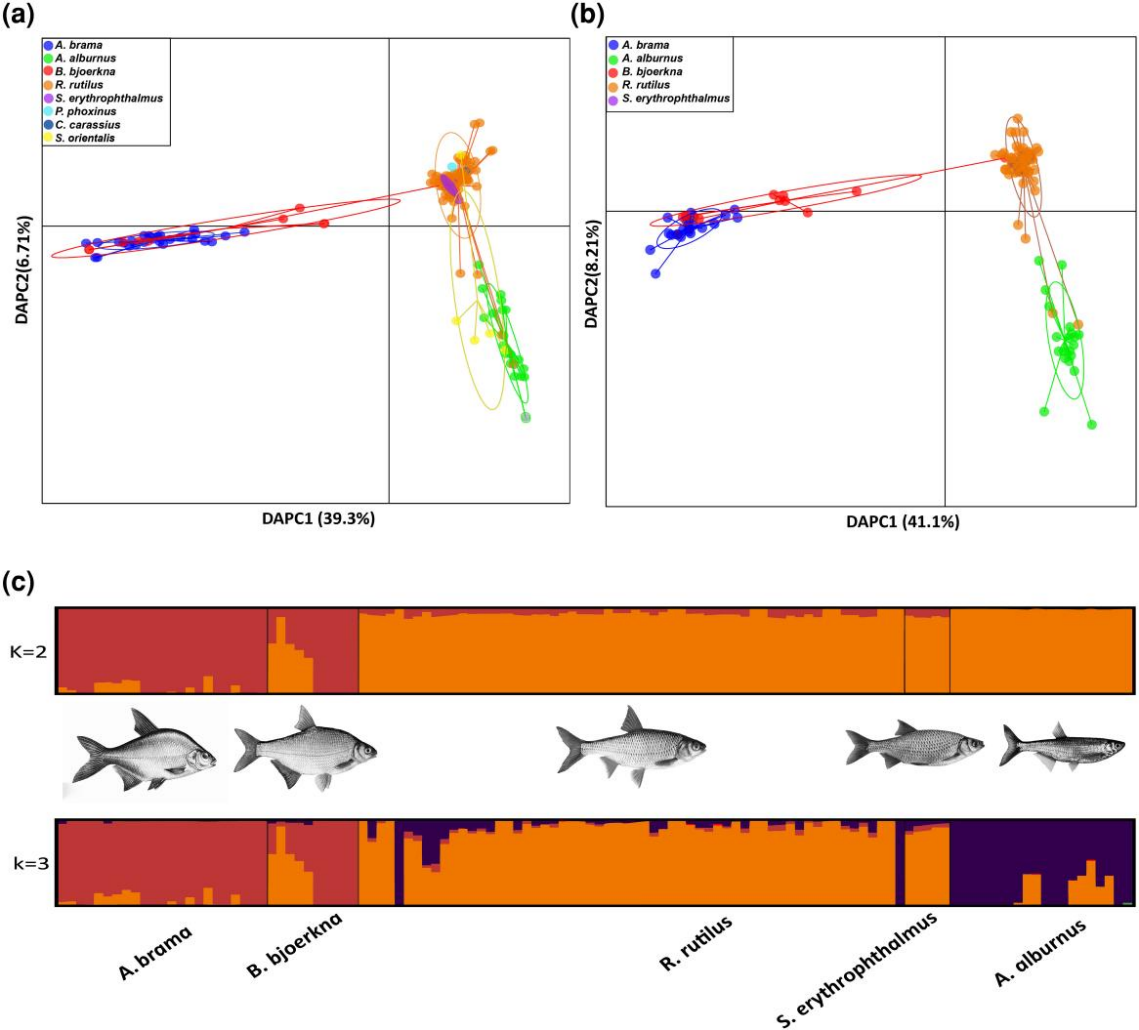
Genetic structure of *Ligula* populations within Lineage A across different fish host species. a) DAPC analysis of datasets B (global, 145 samples) reveals two primary genetic clusters, with the primary axis (DAPC 1) accounting for 39.3% of variance. This axis predominantly segregates populations from *A. brama* and *B. bjoerkna* from other species such as *A. Alburnus, S. erythrophthalmus*, *R. rutilus*, *S. orientalis*, *P. phoxinus*, and *C. carassius*. The secondary axis highlights differentiation between *S. orientalis* and *A. alburnus* versus *R. rutilus* and *S. erythrophthalmus*, contributing to 6.71% of total variance. b) DAPC analysis of dataset D (Czechia-specific, 118 samples) shows a genetic structure similar to the global scale. c) Admixture analysis (dataset D) corroborates the DAPC findings, particularly noting significant admixture in *B. bjoerkna* populations, which exhibit 46% to 90% genetic overlap with the *R. rutilus/S. erythrophthalmus* parasite populations.

To identify the genetic structure and host associations of *L. intestinalis* Lineage A, we applied both nonmodel-based and model-based approaches. First, DAPC analysis of Dataset B (spanning all geographic locations) and Dataset D (specific to Czechia) consistently identified two primary genetic clusters within Lineage A (Fig. 2a and b). This finding was reinforced by the K-means method, which indicated the best partitioning at *K* = 2, as determined by the lowest BIC score (supplementary fig. S2a and b, Supplementary Material online). In Dataset B, which includes parasite populations from all geographic locations, the DAPC analysis (Fig. 2a) revealed two primary genetic clusters along the first axis (DAPC1), which explained 39.3% of the genetic variance. One cluster was associated with parasites from *A. brama* and *B. bjoerkna*, and the other with *A. alburnus*, *S. erythrophthalmus*, *R. rutilus*, *S. orientalis*, *P. phoxinus*, and *C. carassius*. The secondary axis (DAPC2), accounting for 6.71% of the variance, further distinguished parasite populations from *S. orientalis* and *A. alburnus* from those in *R. rutilus* and *S. erythrophthalmus*. In Dataset D, which focuses on Czech populations, the first axis (DAPC1) explained 41.1% of the variance and likewise separated the same two major clusters. The second axis (DAPC2), which explained 8.21% of the variance (Fig. 2b), contributed further to the differentiation of *A. alburnus* from *R. rutilus* and *S. erythrophthalmus* within the second cluster. Admixture analysis of Datasets B and D corroborated the DAPC results, also identifying an optimal *K* = 2, as indicated by cross-validation error (CV, supplementary fig. S2c and d, Supplementary Material online). At a broad geographic scale, parasite populations in Lineage A were divided into two genetic clusters, consistent with the structure also observed at a local scale where all host species co-occur (Fig. 2a and b). The first cluster contained parasite populations from *A. brama* and *B. bjoerkna*, while the second comprised those from *R. rutilus, A. alburnus,* and *S. erythrophthalmus*. Additionally, approximately half of the parasite samples from *B. bjoerkna* exhibited significant admixture, with 46% to 90% assignment to the *R. rutilus/S. erythrophthalmus* parasite cluster (Fig. 2c). At *K* = 3, the parasite population from *A. alburnus* formed a distinct cluster, showing admixture with the population associated with *R. rutilus/S. erythrophthalmus* (Fig. 2c).

**Fig. 3.**
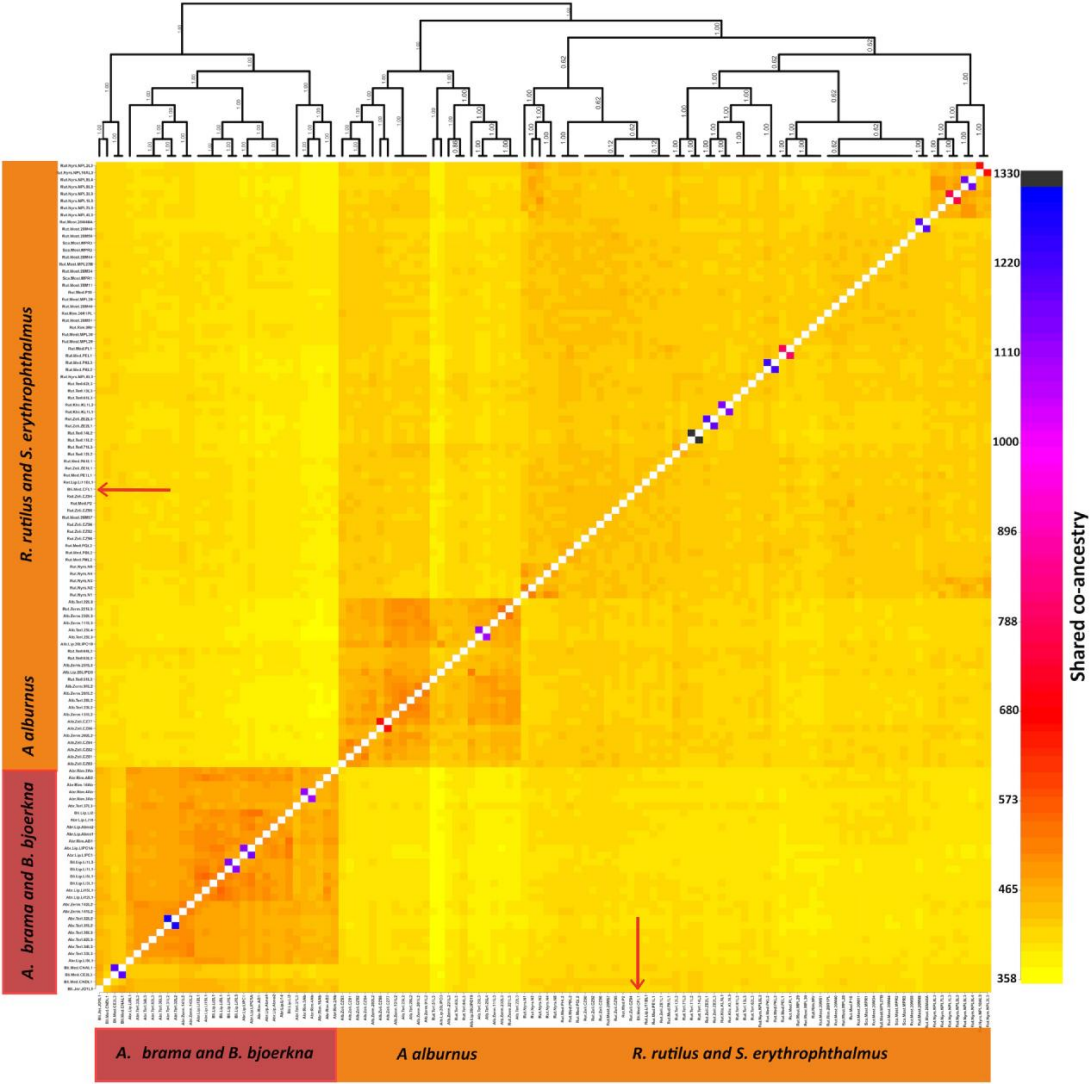
Co-ancestry plot and dendrogram derived from the fineRADstructure evaluation of *Ligula* populations in Czechia. Co-ancestry coefficients transition from low (light color) to high (dark color), indicating the extent of recent shared ancestry between a focal individual (on the vertical axis) and all other individuals included in the study (on the horizontal axis). The arrow highlights a single sample of *Ligula* from the host *B. bjoerkna* (a host switch), which is nested in a genetic cluster from *R. rutilus* and *S. erythrophthalmus*.

Furthermore, fineRADstructure analysis (Malinsky et al. 2018) validated Admixture clustering results (*K* = 2), demonstrating strong support in the dendrogram (Fig. 3). It also showed the parasite population in *A. alburnus* as a sub-cluster within the *R. rutilus*/*S. erythrophthalmus* group, consistent with Admixture analysis results observed at *K* = 3. FineRADstructure highlighted extensive shared co-ancestry between parasite populations in *A. brama* and *B. bjoerkna*, though this cluster exhibited lower co-ancestry with other populations at both local and broad scales (supplementary fig. S3, Supplementary Material online and Fig. 3). In line with the admixture results, one *B. bjoerkna* sample clustered with *R. rutilus/S. erythrophthalmus* parasite populations.

Lastly, the Mantel test revealed no significant correlation between genetic distance and geographic location within Lineage A parasite populations, suggesting that geographic distance does not impede gene flow at the intra-population level (supplementary fig. S4, Supplementary Material online).

### Divergence Modeling of Parasite Populations

**Fig. 4.**
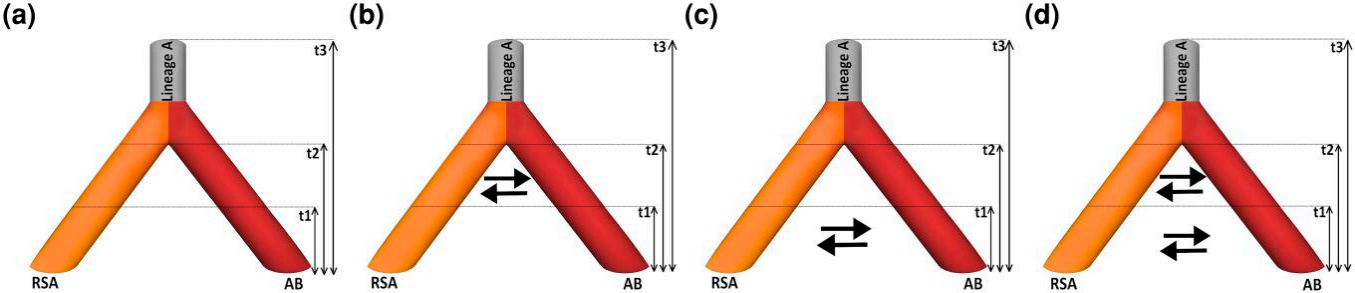
Four speciation scenarios testing historical gene flow and demography patterns in Lineage A. a) Represents the allopatric speciation model with complete geographic isolation indicated by no gene flow between parasite populations from *R. rutilus*, *S. erythrophthalmus*, and *A. alburnus* (RSA) and parasite populations from *A. brama* and *B. bjoerkna* (AB). b) Depicts the model with post-divergence gene flow through primary contact. c) Shows post-divergence speciation with secondary contact leading to recent gene flow. d) Represents the scenario with continuous gene flow, as indicated by multiple gene flow arrows between populations over time.

We tested four speciation models to assess parasite population divergence: allopatric speciation without gene flow, isolation with primary contact (divergence accompanied by continuous gene flow from the initial split), secondary contact (initial divergence without gene flow followed by recent renewed gene flow), and sympatric divergence with continuous gene flow (ongoing gene flow throughout divergence) (Fig. 4). Model comparisons revealed that the most likely scenario involved isolation with ongoing gene flow, where gene flow rates have increased in recent generations. The change in the rate of gene flow was inferred to have occurred about 58,000 generations ago. However, these time estimates should be interpreted with caution as we fixed the first split to the lower bound of 160,000 generations ago (corresponding roughly to the time since when two parasite populations probably diverged from each other (Nazarizadeh et al. 2023). Splitting times may be even more recent if there was a significant time lag between gene tree and species tree. The second most likely model, with a ΔAIC of 28.1, suggested recent gene flow starting 9,100 generations ago, representing secondary contact. Models involving primary contact showed very close likelihoods (ΔAIC 25.4), while the model excluding gene flow was a poor match for the data (ΔAIC 96.4). All ΔAIC values are calculated relative to the best-supported model, which had the lowest AIC. These findings indicate speciation occurred amidst gene flow. However, having only two migration matrices is a strong simplification of the speciation process (supplementary table S2, Supplementary Material online).

### Gene Flow Among Parasite Populations in Different Hosts

**Fig. 5.**
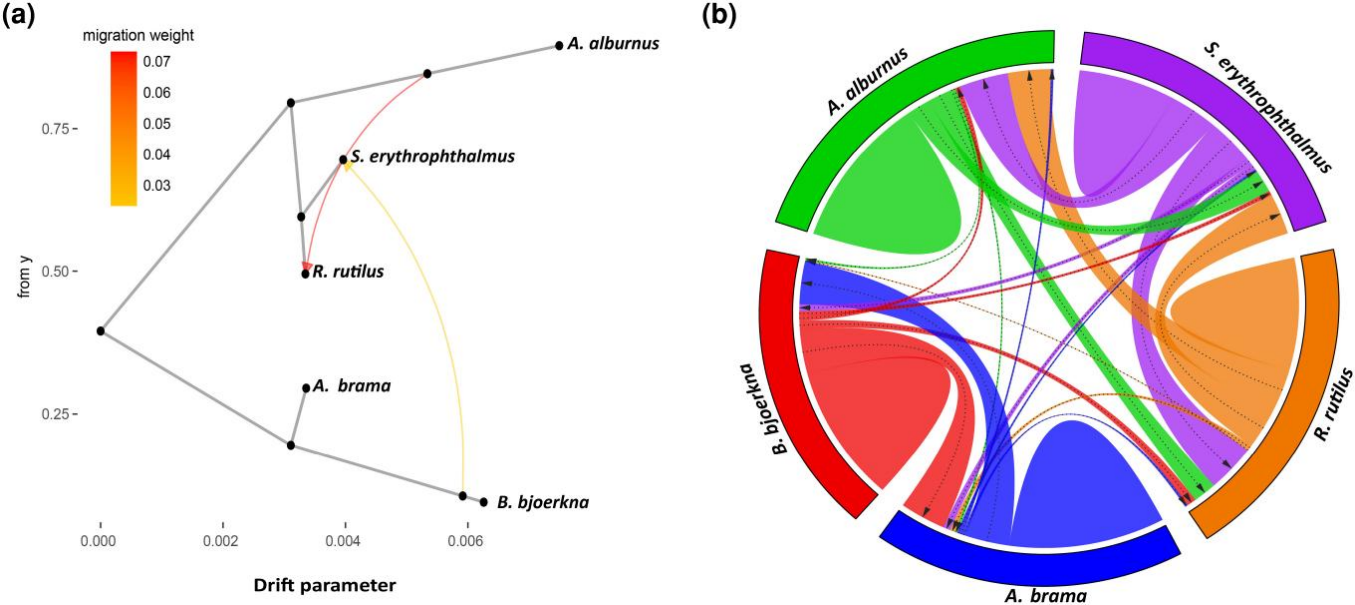
Analysis of gene flow among *Ligula* populations infecting sympatric fish hosts. a) Species tree derived from Treemix shows two principal genetic clusters with significant gene flow between (*B. bjoerkna* and *S. erythropthalus* hosts) and within them (*A. Alburnus* and *R. rutilus* hosts). b) Contemporary gene flow reveals predominant within-host exchange, with clear symmetrical contributions between *A. brama* and *B. bjoerkna*. Reciprocal gene flow is also observed among *R. rutilus*, *A. alburnus*, and *S. erythrophthalmus*, indicating ongoing cross-host connectivity.

To explore historical and contemporary gene flow among sympatric *L. intestinalis* populations, we analyzed Dataset D (unlinked SNPs from Czech samples) using Treemix (Pickrell and Pritchard 2012) to infer past migration events and BA3SNP (Mussmann et al. 2019) to assess recent gene flow patterns. The unrooted species tree derived from the Treemix analysis identified two primary genetic groups (Fig. 5a), corroborating the findings from both the admixture and fineRADstructure analyses. Notably, the data are best explained by a model that considers two migration events. Parasite populations in *A. brama* and *B. bjoerkna* demonstrated a close genetic relationship, forming a sister lineage distinct from other parasite groups. The most prominent migration events were observed between the *A. alburnus* and *R. rutilus* parasite populations. Additionally, a gene flow with a lower weight was detected between *B. bjoerkna* and *S. erythrophthalmus* samples (Fig. 5a). The results of the contemporary gene flow (Fig. 5b) indicated that most of the gene flow occurred in populations within the same host species. While there was some gene flow between different hosts, populations in *A. brama* and *B. bjoerkna* symmetrically contributed to the pool of exogenous allelic variants. Similarly, reciprocal gene flow was observed among the parasite populations in *R. rutilus*, *A. alburnus*, and *S. erythrophthalmus*, with comparable levels of exchange in both directions. Lastly, small level of gene flow was detected between populations in *A. brama* and *B. bjoerkna* and those in *A. alburnus*, *R. rutilus*, and *S. erythrophthalmus* (Fig. 5b).

### Detection of SNPs Under Divergent Selection

**Fig. 6.**
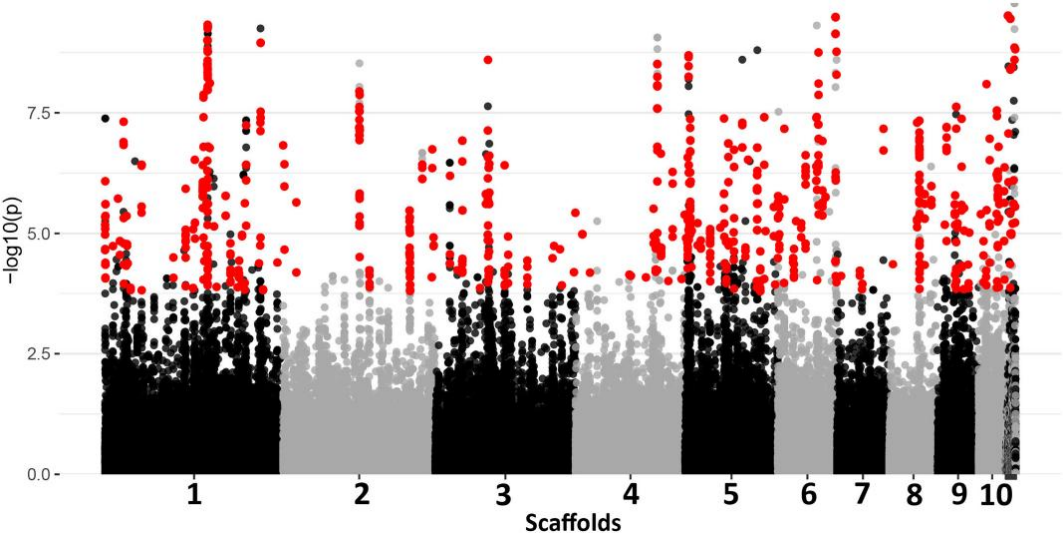
A consensus Manhattan plot displaying results from four genome-wide selection analyses: PCAdapt, Outflank, BayeScan, and HapFLK. This plot presents the 10 longest scaffolds of the *Ligula* genome (additional scaffolds are also included in the plot and are represented collectively due to their shorter lengths). Highlighted SNPs indicate those under selection between two parasite populations found in sympatric hosts. The *y*-axis shows −log10(*p*) values derived from hapFLK.

Using Dataset C (all Czech samples and all SNPs), selection analyses were conducted to identify loci potentially under divergent selection in two parasite populations structured within sympatric hosts. A total of 3,004 outlier loci, putatively under selection, were identified using PCAdapt, OutFLANK, BayeScan, and HapFLK. The least conservative method, BayeScan, identified the highest number of outliers with 2,367 SNPs, PCAdapt detected 1,743 SNPs, whilst OutFLANK and HapFLK were the most conservative, pinpointing 1,384 and 1,256 loci, respectively. Notably, 896 SNPs (representing 29.8% of the total outlier SNPs) were detected by all four methods, collectively constituting the putatively adaptive dataset (supplementary fig. S4, Supplementary Material online). Most of the commonly detected loci were found in the 10 longest scaffolds of the *Ligula* genome (Fig. 6). Out of the 896 outlier loci, 156 were directly associated with the coding sections of 65 gene models in the *Ligula* genome (supplementary table S3.1, Supplementary Material online). Considering potential gaps in the genome annotation, a second method was applied, extending the search to 100 bp before and after each outlier. This approach revealed 37 additional outliers, sharing homology with genes from closely related tapeworm species of the family Diphyllobothriidae (supplementary table S3.1, Supplementary Material online).

**Fig. 7.**
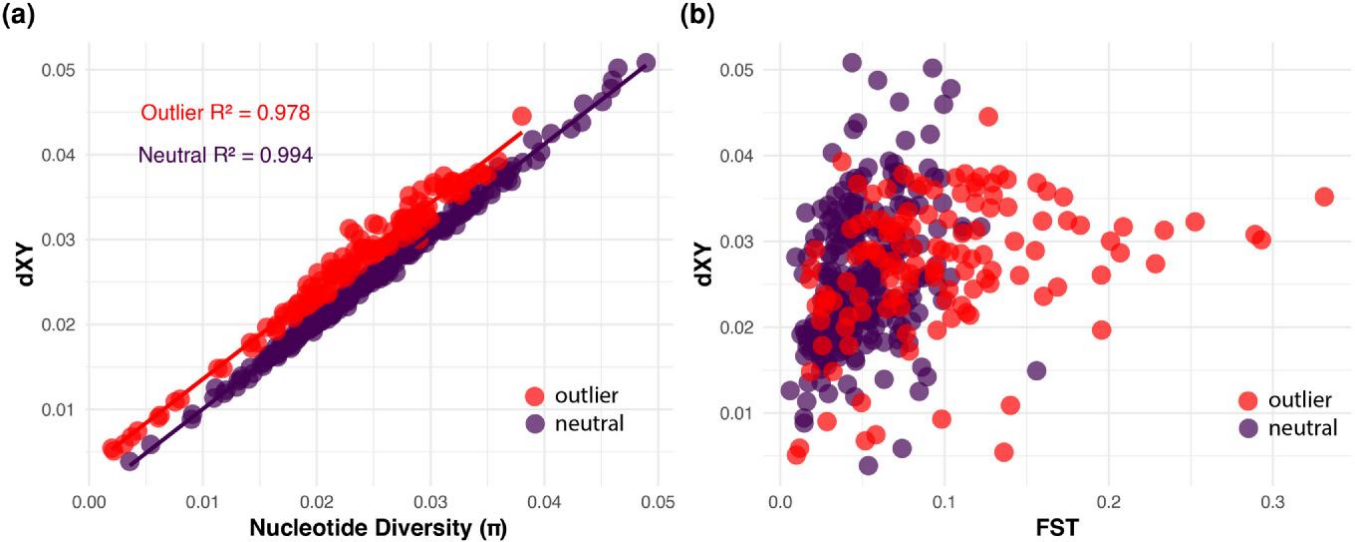
Comparative analysis of genome-wide variations between putative outlier and nonoutlier loci. a) Correlation between dXY and pi distinguishing outlier from neutral loci. b) Patterns of dXY distribution in association with FST, highlighted for both outlier and neutral loci.

A significant positive correlation was observed between the values of absolute genetic divergence (dXY) and nucleotide diversity for both neutral and outlier loci (Fig. 7a). Outlier loci predominantly displayed a higher dXY compared with neutral loci, at similar levels of nucleotide diversity, and exhibited lower to moderate nucleotide diversity in contrast to neutral loci, for comparable dXY values (Fig. 7a). Similarly, dXY showed a positive correlation with *F*_ST_, and outlier loci displayed a relatively higher *F*_ST_ compared with neutral loci (*R*^2^ = 0.978 for outliers, *R*^2^ = 0.994 for neutral loci) (Fig. 7b). Notably, the distributions of Tajima's *D* revealed significant negative values for putative outlier in comparison to nonoutlier loci (*P* value < 0.01, supplementary fig. S5, Supplementary Material online).

### Differential Gene Expression

**Fig. 8.**
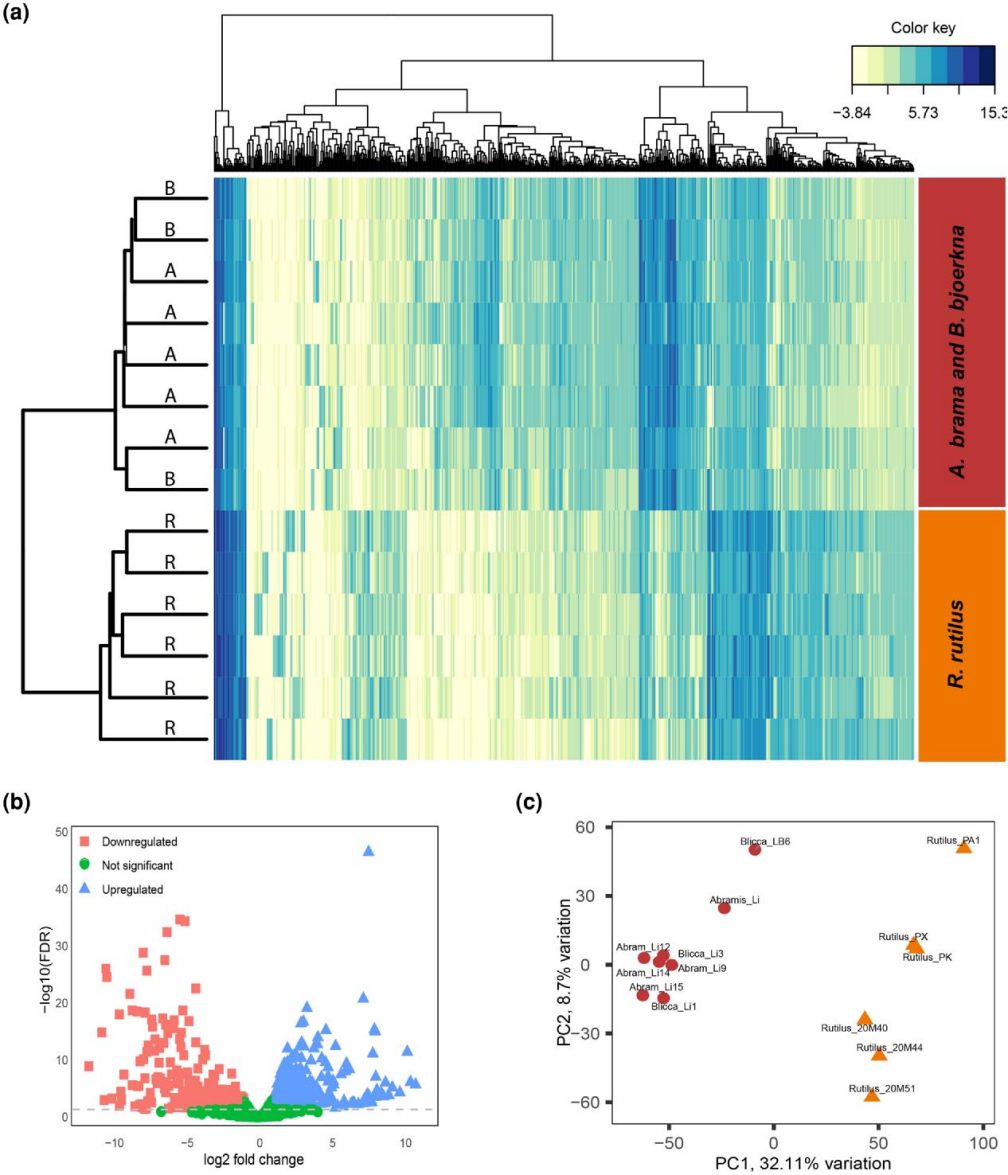
DGE in *L. intestinalis* samples obtained from three host species living in sympatry. a) Heatmap illustrates the differential expression of genes across parasite populations in different host species. The dendrogram on the left highlights sample clustering, with parasite samples labeled as “A” for *A. brama*, “B” for *B. bjoerkna*, and “R” for *R. rutilus*. b) Volcano plot displaying upregulated (rectangles), downregulated (triangles), and nondifferentially expressed transcripts (dots) across hosts (*R. rutilus*, *A. brama,* and B. *bjoerkna*). c) PCA showcasing the clustering of sample replicates, with relative variances detailed across PC1 and PC2.

To understand how host-associated divergence influences gene expression patterns, we analyzed transcriptome profiles of parasite populations infecting different sympatric fish hosts (Fig. 8). The transcriptome samples used in this analysis represent biological replicates from parasite populations belonging to two distinct genetic clusters associated with three sympatric host species: *R. rutilus* (six samples), *A. brama* (five samples), and *B. bjoerkna* (three samples) (see supplementary table S1, Supplementary Material online). Gene expression analysis of 14 transcriptome samples derived from three different hosts in a sympatric setting, identified 993 DEGs: 556 were up-regulated and 367 down-regulated in the parasite populations of *R. rutilus* compared with *A. brama* and *B. bjoerkna* (supplementary table S3.S2, Supplementary Material online, Fig. 8). The RNA expression profiles showed that parasites in two of the hosts, *A. brama* and *B. bjoerkna*, had similar transcriptome patterns. In contrast, parasites in *R. rutilus* showed a distinct transcriptomic profile (Fig. 8a). Furthermore, the parasite population in *R. rutilus* differed from those in *A. brama* and *B. bjoerkna* in the first principal component (PC1) of the PCA, which accounted for 32.11% of the observed variance (Fig. 8c). This pattern is consistent with the results of the genetic structure of the parasite populations.

### Gene Ontology Patterns in DEGs and SNPs Under Selection

**Fig. 9.**
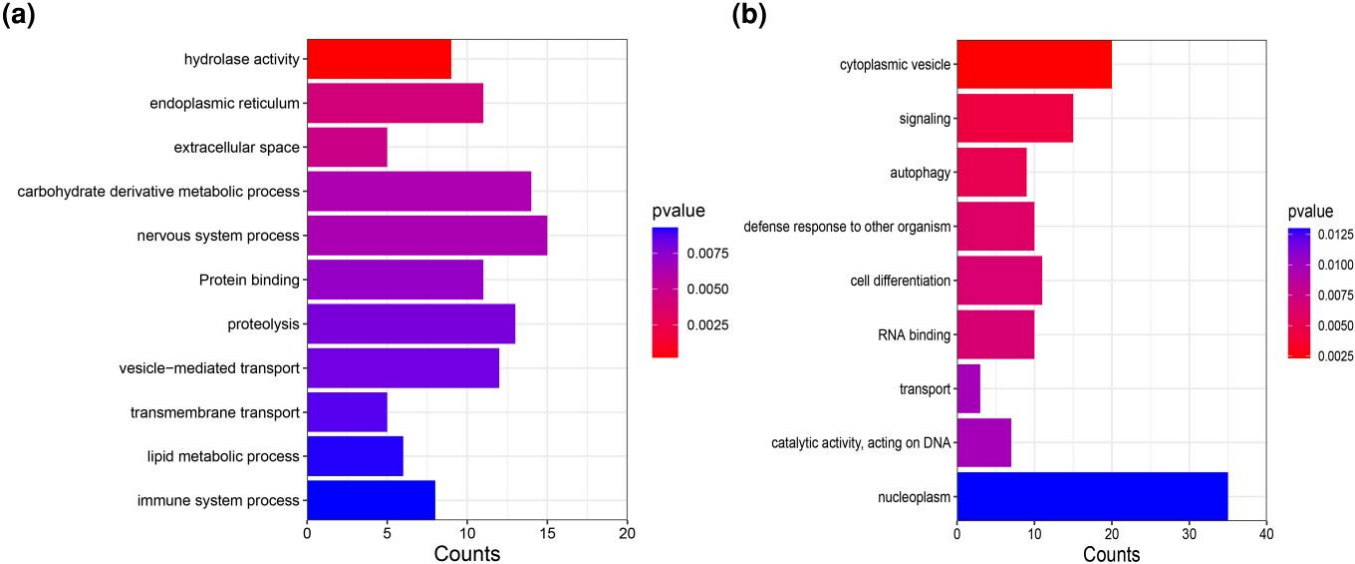
Differential GO term enrichment in *L. intestinalis* clusters parasitizing different hosts. a) Overrepresented GO terms in the parasite population from *R. rutilus*, highlighting key biological processes, CC, and MFs with the respective gene counts. b) Overrepresented GO terms in parasite populations from *A. brama* and *B. bjoerkna* hosts, showcasing the distinct adaptive molecular responses and interactions within these environments. The *y*-axis represents the number of genes associated with each GO term.

The results of the Gene Ontology (GO) analysis revealed significant variations in gene enrichment in parasite populations across different host environments (supplementary table S3.3, Supplementary Material online, Fig. 9). In the parasite population from *R rutilus*, the Biological Process (BP) ontology was highlighted by dominant terms such as “nervous system process” with 15 genes and “proteolysis” with 13 genes, indicating a possible focus on neural activities and protein degradation. Regarding Cellular Components (CC), genes associated with “extracellular space” and “endoplasmic reticulum (ER)” were particularly prominent, suggesting interactions between cellular organelles and the external environment. The Molecular Function ontology (MF) highlights the significant presence of “hydrolase activity” in this environment, indicating central enzymatic activities (Fig. 9a). In contrast, in *L. intestinalis* populations from *A. brama* and *B. bjoerkna*, the BP ontology showed “signaling” with 15 genes (Fig. 9b), indicating important communication and regulatory pathways. The CC ontology was dominated by “nucleoplasm” with 35 genes, indicating potentially enhanced nuclear activities or regulations. “Cytoplasmic vesicles” also played an important role, indicating important intracellular transport mechanisms. Within the MF ontology, there was a notable focus on “RNA binding” and “Catalytic activity acting on DNA”, indicating essential molecular interactions and potential DNA manipulations.

The ddRAD-derived putative outlier data revealed a comprehensive profile of GO term enrichment within *L. intestinalis* populations. In total, 291 unique GO terms were identified (supplementary table S3.1, Supplementary Material online). Of these, the BP ontology included 129 enriched terms (44.3%), highlighting key cellular and physiological functions such as autophagy, DNA integration, response to abiotic stimulus, generation of precursor metabolites and energy, regulation of signaling, immune system processes, and ammonium ion metabolic process (supplementary fig. S7, Supplementary Material online). The CC ontology featured 73 enriched terms (25.1%), emphasizing subcellular structures such as the cytosol, lipid droplet, sarcolemma, and plasma membrane (supplementary fig. S8, Supplementary Material online). Although the MF ontology showed the fewest terms (53, 18.2%), it encompassed a functionally diverse set of biochemical activities, including cAMP binding, ether hydrolase activity, protein binding and catalytic activity (supplementary fig. S9, Supplementary Material online). A comparative analysis with previously evaluated transcriptome data revealed a common enrichment of GO (Nazarizadeh et al. 2024). These common terms from proteolysis, nucleolysis, immune system processes, signaling, cytosol, and hydrolase activity highlight the consistent molecular and cellular activities central to *L. intestinalis* populations across different analytical platforms and datasets. Overall, five genes were identified (ANN07147, ANN10664, ANN13534, ANN02916, and ANN12081) as common between the ddRAD selection analyses and DGE analysis.

## Discussion

The interplay between ecological pressures and natural selection in shaping host specificity is a critical factor in the evolutionary dynamics of parasites (Simmonds et al. 2020; Bellis et al. 2022; Nikolakis et al. 2022). In the present study, we have employed genome-wide SNPs and transcriptome data to unravel the complexities of the intraspecific population structure within a single evolutionary lineage of *L. intestinalis* tapeworm. This study places a special focus on the parasite's interactions with its second intermediate hosts, thus shedding light on how ecological factors play a significant role in driving speciation between closely related parasite populations. Our research was propelled by the intriguing observation of two distinct parasite populations coexisting in sympatry. Notably, these two clusters exhibited a high level of concordance with two different groups of host species, suggesting a subtle yet significant interplay between host specificity and the evolutionary trajectory of the parasite. Our study suggests that the genetic divergence between the two parasite clusters is indicative of speciation with ongoing gene flow, where disruptive selection facilitates reproductive isolation. Direct association between ecological divergence and reproductive isolation is a keenly sought phenomenon in specialist organisms, including parasitic plants (Giraud et al. 2010), parasitic wasps (Stelinski and Liburd 2005), leaf mites (Skoracka et al. 2013) and phytophagous insects (Berlocher and Feder 2002; Funk et al. 2002). However, only a few studies explored this link using genomic data and in a framework including ongoing gene flow (Hume et al. 2018; Villacis-Perez et al. 2021). Below, we discuss the results of our investigation, examining the role of ecological speciation in the evolution and diversification of this parasite.

### Population Genetic Structure and Divergence With Ongoing Gene Flow

Analysis of genetic structure using genome-wide SNP data revealed that *L. intestinalis* Lineage A is divided into two primary clusters within its Palearctic distribution. At this intraspecies level, distinct differences were observed in the parasite populations of *A. brama* and *B. bjoerkna* compared with those in *R. rutilus*, *S. erythrophthalmus*, *A. alburnus*, *P. phoxinus*, and *C. carassius*. This pattern was also evident at a more local scale within Czechia, where these host species were sampled in sympatry, highlighting the role of host specificity in the parasite's evolution. These findings align with our previous study that revealed a nonrandom sharing of a mtDNA haplotype between parasite individuals from *A. brama* and *R. rutilus* (Nazarizadeh et al. 2022). Yet, earlier investigation involving more individuals showed a high number of shared mtDNA haplotypes and an indistinguishable population structure among all parasite populations in Lineage A (Bouzid et al. 2008b). This result indicates that while mtDNA provides valuable insights into broad evolutionary patterns and phylogeography, it may be less effective in detecting subtle, microevolutionary changes and ecological divergence that occur over short timescales or in complex population structures. In contrast, genome-wide SNP and nuclear marker analyses offer a more comprehensive and detailed view of these processes.

Ecological divergence often interacts with gene flow, the extent of which varies with dispersal distance (Räsänen and Hendry 2008). In our study, we observed genetic divergence among parasites from two related genetic clusters, yet we also identified notable gene flow between them. Our Treemix analysis revealed two major groups of parasite populations: the first cluster associated with three hosts (*A. alburnus*, *R. rutilus*, and *S. erythrophthalmus*), and the second cluster with two hosts (*B. bjoerkna* and *A. brama*). Within the first cluster, significant gene flow was detected between parasites infecting *A. alburnus* and *R. rutilus*. Additionally, gene flow between the two major clusters was identified, specifically between the parasites from *B. bjoerkna* and *S. erythrophthalmus*. This pattern was further supported by admixture analysis, which identified hybrid individuals originating from parasite populations infecting *B. bjoerkna* and *R. rutilus/S. erythrophthalmus*. Our results are in line with the contemporary perspective of sympatric speciation in the genomic era, in which gene flow is almost pervasive between recently diverged sister lineages (Marques et al. 2019b).

This admixture likely indicates cross-infections in the definitive host, piscivorous bird, where reproduction takes place. Hybrid progeny arises when different parasite clusters infect the same bird individual. Previous studies recorded sympatric occurrence of several *Ligula* strains in the same host species (Bouzid et al. 2008a). Despite this opportunity for recombination in the definitive host, the predominant genetic pattern in Lineage A *Ligula* still clearly reflects host affiliation of the parasite progeny in the second intermediate hosts (cyprinid fish). This suggests that, although gene flow at least occasionally occurs at the adult stage in birds, strong fish-host associated selection during the plerocercoid stage maintains ecological divergence. Based on our population structure results it seems that highly admixed individuals survive only in the *B. bjoerkna* host and are not present in others. This partial permeability of *B. bjoerkna* for the other cluster is reflected by the presence of a single individual belonging to the *R. rutilus*/*S. erythrophthalmus* cluster in this host. Experimental evidence from related systems supports our selection-based interpretation; for example, hybrids between *Schistocephalus solidus* and *S. pungitii* can be successfully cultured both in vitro and within the bird host, yet such hybrids are rarely detected in natural populations of the second intermediate fish hosts (sticklebacks) (Henrich et al. 2013; Henrich and Kalbe 2016). Therefore, the observed genetic structure is likely shaped by both gene flow via the definitive host and selection for host specialization in the fish hosts.

We aimed to distinguish between historical secondary contact and ongoing gene flow in the *Ligula* system by analyzing patterns of genetic diversity at outlier loci and testing demographic scenarios. We found a strong positive correlation between dXY and π across both outlier and background loci, a pattern generally consistent with neutral divergence rather than divergence with gene flow (Cruickshank and Hahn 2014). However, a subset of outlier loci showed elevated dXY relative to neutral loci at similar π levels, suggesting that some genomic regions may have resisted introgression, potentially due to divergent selection. These outlier loci also tended to exhibit lower to moderate nucleotide diversity, consistent with directional selection reducing within-population variation (Nosil et al. 2009). The relationship between dXY and *F*_ST_ was more scattered, with several outliers showing high *F*_ST_ but only moderate dXY a pattern that supports the hypothesis that elevated *F*_ST_ can result from reduced diversity rather than from barriers to gene flow (Cruickshank and Hahn 2014). This interpretation is further supported by negative Tajima's *D* values at outlier loci, indicative of recent selective sweeps. Therefore, these genomic signals suggest that divergent selection, rather than genome-wide barriers to gene flow, is driving localized divergence. This pattern aligns with our demographic modeling results, which support continued gene flow in the face of selection-driven differentiation, a dynamic that can facilitate ecological speciation even in the presence of connectivity between populations.

Multiple studies in free-living species (e.g. Momigliano et al. 2017; Teske et al. 2019) highlight the potential influence of gene flow and disruptive selection in the process of speciation, leading to the divergence of a single ancestral population into groups that specialize in different habitats, while the evidence in co-evolving species is more scarce (e.g. Appelgren et al. 2018; Simmonds et al. 2020). Our historical divergence modeling revealed that the most plausible evolutionary scenario for the divergence between parasite clusters is speciation through isolation with migration. This involves recurrent migration after divergence, as illustrated in Fig. 4d. The divergence of these two parasite clusters likely occurred in the climatically unstable Chibanian period (Middle Pleistocene, according to Nazarizadeh et al. 2023), possibly facilitating the colonization of and host shifting among various cypriniform hosts. Host switches can be followed by ecological disruptive selection enhancing the fitness of each population within its respective host groups. In such scenario, segments within a parasite population begin to specialize in exploiting different host species sympatrically, within the same ecosystem. Then, one subgroup of a parasitic species might adapt to thrive with a specific host, exploiting unique physiological or immunological traits of that host, while another subgroup might specialize in a completely different host species.

### Candidate Loci and Transcriptome Genes Involved in Adaptation to Host

Detection of significant genetic differentiation and selection-related outlier SNPs implies that changes in gene expression might be attributed to evolutionary adaptations, rather than just to plastic responses to varying environments (Mathieu-Bégné et al. 2022). In this study, we explored the genomic differentiation between two parasite populations living in sympatric hosts over different time scales by comparing genomic outlier loci indicative of long-term adaptation with DEGs representing short-term acclimation. Although our transcriptome data did not cover all parasite populations from the five hosts studied, our results were consistent with genome-wide SNP analyses, revealing clear separate profiles of gene expression between parasite populations from *R. rutilus* and those in *A. brama* and *B. bjoerkna*. These differences in gene expression profiling between the two host groups are associated with various biological tasks. We will further explore possible explanations for the observed DEG patterns in relation to the parasite's adaptation to different hosts. Yet, it should be noted that our field-based transcriptome approach inherently involves environmental variability, which may influence observed gene expression patterns. Moreover, the application of enrichment analysis to *L. intestinalis* can be challenging due to difficulties in assigning orthology, which is essential for transferring functional information. This challenge arises primarily from the large phylogenetic distance between *Ligula* and other model organisms within the class Cestoda, coupled with the rapid molecular evolution of the parasites (Morand 2000). Consequently, GO may yield uncertain functional inferences.

Our findings include five genes showing a direct correlation between outlier loci and DEGs, either in terms of gene identity or physical proximity, along with notable functional parallels. Of these five DEGs, ANN02916 and ANN12081, are associated with reverse transcription activities. Aligning with a genome description by Nazarizadeh et al. (2024), ANN02916 was identified as significantly downregulated during the transition from the larval to the adult stage in *L. intestinalis*. The reverse transcription process involves DNA sequences in an organism's genome derived from RNA, which is a key process in retroviruses and retrotransposons (Hughes 2015). Additionally, enrichment analyses revealed an overrepresentation of genes linked to immune system processes in the *Ligula* population from *R. rutilus* (Fig. 9a). These findings suggest that DNA integration and reverse transcription activities may play crucial roles in shaping host specificity and immune evasion strategies of the parasite. Several fish studies (Ottová et al. 2005; Šimková et al. 2021, 2023) indicated that the Major Histocompatibility Complex profiles of two hosts, *A. brama* and *R. rutilus* (representing two divergent parasite genetic clusters), are significantly different. Therefore, the two parasite populations are likely confronted with distinct immune profiles, driving differential adaptations and immune evasion strategies.

The three other genes, ANN07147, ANN10664, and ANN13534, identified as significant in both SNPs under selection and DEGs play critical roles in biological functions linked to the ER membrane and transmembrane transport. Both processes may be important for the survival and host specificity of parasites, the membrane is key for carrier-mediated transport within the tapeworm tegument, greatly influencing the chemical modification of absorbed substances and offering protection against the host's digestive enzymes. Notably, this membrane includes a glycocalyx layer, containing oligo- or polysaccharide, which is fundamental for binding various substances, such as inorganic ions and larger organic molecules including host enzymes. While some of these enzymes remain active on the worm's surface, facilitating contact digestion, others are bound in a nonactive form, possibly serving as a defence mechanism to prevent the parasite from being digested by the host. Additionally, our results highlight the biological function of glycosylation, which is potentially linked to the maintenance of the glycocalyx layer of the tegument (Hildreth et al. 1997). The presence of glucose transporter homologs in *Taenia solium* suggests a link between glucose absorption through the tegument and glycosylation processes (Rodríguez-Contreras et al. 1998). Glycosylation is not only crucial for the tapeworm's nutrient absorption but also plays a significant role in host-parasite interactions. Antigenic properties of glycolipids in *Spirometra erinaceieuropaei* are a prime example (Yanagisawa et al. 1999). Additionally, the presence of glycoconjugates in the tegument of *Taenia taeniaeformis* may contribute to the parasite's resistance to host digestive processes (Olson et al. 2000).

In conclusion, using whole-genome genotyping and transcriptome analyses, we demonstrated how host-specialization leads to sympatric genetic differentiation in spite of continued gene flow. We proposed disruptive host-mediated selection on genes involved in immunological or physiological interaction with the host as the driver of ecological speciation in this pathogenic parasite of freshwater fish. This study provides an example of a frequently anticipated, but rarely observed, ecological phenomenon of speciation by host specificity.

## Materials and Methods

### Sample Collection

We collected a total of 83 plerocercoid samples of *L. intestinalis* from five prevalent host species in Czechia: *R. rutilus*, *A. alburnus*, *B. bjoerkna*, *S. erythrophthalmus*, and *A. brama* (Nazarizadeh et al. 2022). These specimens were sourced from 10 different freshwater ecosystems throughout Czechia (see supplementary table S1, Supplementary Material online and Fig. 1) using gillnets, in the frame of a previous hydrobiological research (see Nazarizadeh et al. 2022 for sampling details). The samples were fixed in 96% ethanol and stored at a refrigerated temperature prior to DNA extraction. For the purpose of transcriptome sequencing, we specifically selected 1-year-old fish harboring a single tapeworm, all of which were collected within a 2-week period during summer 2022, to standardize conditions and minimize transcriptome expression variability. This led to collection of 14 samples from three key host species (*R. rutilus*, *B. bjoerkna*, and *A. brama*). Additionally, the time between fish capture, dissection, and sample fixation was standardized to reduce stress-induced transcriptional changes and ensure comparable handling protocols. The central segment of each tapeworm was sectioned into several 5×5 mm squares, which were then submerged in RNAlater (Ambion, Austin, Texas, USA) and maintained at 4 °C overnight to stabilize and protect the RNA, and then stored at −80 °C until RNA extraction.

### DNA Extraction and ddRAD Library Preparation

Total genomic DNA was extracted from specimens preserved in 96% ethanol using the DNeasy Blood and Tissue Kit (Qiagen). The quality and quantity of the DNA samples were verified using a 0.8% agarose gel and a Qubit 2.0 Fluorometer, respectively. Double digest restriction-site associated DNA (ddRAD) libraries were generated using the nspI + mluCI enzymes and a modified ddRAD protocol established by Peterson et al. (2012), employed to generate ddRAD data for our previous *L. intestinalis* study (Nazarizadeh et al. 2023). All samples were pooled into two libraries, which were then sequenced in two lanes to maintain uniform coverage. This process generated paired-end reads of 150 bp on an Illumina NovaSeq (Novogene UK), yielding approximately 6.7 million paired-end reads per sample. The procedures for assembling multiplexed ddRAD-seq libraries for each barcoded individual sample (supplementary table S1, Supplementary Material online), as well as the detailed purification processes, were adopted from Nazarizadeh et al. (2023).

### Transcriptome Sequencing

To compare the transcriptome profiles among different parasite populations, we extracted RNA from plerocercoid samples following the method of Chomczynski and Sacchi (Chomczynski and Sacchi 1987), utilizing the acid guanidinium thiocyanate-phenol-chloroform procedure with reagents from Invitrogen (Carlsbad, CA, USA). The RNA yield was estimated using the Qubit RNA Broad Range Assay Kit (Thermo Fisher), and its integrity was assessed with an Agilent Bioanalyzer 2100 (Agilent Technologies, USA). Subsequently, the RNA samples underwent commercial processing to be converted into cDNA libraries, followed by sequencing using the Illumina NovaSeq 150 PE read technology facilitated by Novogene (UK).

### ddRAD Data Assembly and SNP Calling

We applied the process_radtags program from Stacks v.2.5.6 (Rochette et al. 2019) to demultiplex and filter out raw reads with low-quality or unidentified bases from the 83 Czech samples. To enhance our dataset, we also integrated ddRAD data for another 62 Lineage A individuals, derived from an earlier phylogeographic study on *L. intestinalis* (Nazarizadeh et al. 2023; see supplementary table S1, Supplementary Material online). Subsequently, all paired-end reads were aligned to the *Ligula* reference genome (BioProject PRJNA1055111; Nazarizadeh et al. 2024) using Bowtie v2.5.0's default settings (Langmead and Salzberg 2012). We then sorted and converted all alignments to BAM format using Samtools v1.18 (Danecek et al. 2021) and conducted reference assembly for genotype calling and locus assembly using the ref_map.pl wrapper in Stacks. Different filtering options were applied to the genotype calling process (detailed in the supplementary text S1, Supplementary Material online) resulting in the generation of four distinct SNP matrices in VCF format: **Dataset A**, which incorporates all samples from Lineage A (145 samples), featuring 334,468 SNPs with an average locus coverage of 21×; **Dataset B**, containing 39,127 SNPs/loci after LD filtering across all Lineage A individuals, with an average locus coverage of 18×; **Dataset C**, developed exclusively for the samples from Czechia to focus at populations in sympatry (118 individuals), including 379,621 SNPs with a 21× average locus depth of coverage; and **Dataset D**, comprising only unlinked SNPs from the Czech samples (72,696 SNPs/loci) with an average locus coverage of 17.

### Genome-wide Diversity

We used the R package SambaR v 1.10 (de Jong et al. 2021) to compute several metrics of genetic diversity—including observed heterozygosity (Ho), expected heterozygosity (He), standardized multilocus heterozygosity (sMLH), Watterson's theta (θ_W_), and the inbreeding coefficient (Fis)—across different parasite populations, using Dataset A, which contains 334,468 SNPs.

### Population Genetic Structure

To understand the impact of host specificity on the population genetic structure of parasites, we employed three genetic clustering methods on two datasets: Lineage A samples (Dataset B) and Czechia-specific samples (Dataset D). First, we performed a discriminant analysis of principal components (DAPC) using the R package adegenet (Jombart 2008). Next, we implemented the ADMIXTURE algorithm with 20 replicates per *K* value (1 to 7) in AdmixPiPe v3 (Mussmann et al. 2020), choosing the best K through the lowest CV error and visualizing the results in CLUMPAK (http://clumpak.tau.ac.il/). Finally, we employed fineRADstructure and RADpainter v.0.2 (Malinsky et al. 2018) to assess nearest-neighbour haplotypes, allowing a maximum of 10 SNPs per locus and 25% missing data during haplotype file conversion, extending burn-in iterations to 200,000 with 1,000,000 total iterations to ensure MCMC convergence, and generating co-ancestry heatmaps using the “FinestructureLibrary.R” function. Detailed methodologies for these analyses are provided in the supplementary text S2, Supplementary Material online.

### Coalescent Analysis of Speciation Modeling

To better understand the impact of isolation and migration on population differentiation within Lineage A, we utilized a coalescent-based approach implemented in fastsimcoal v2.7.9.3 (Sousa and Hey 2013; Excoffier et al. 2021) to examine gene flow and demographic history. Four speciation models were proposed, and their corresponding demographic models were applied to two parasite populations, identified by the analysis of population genetic structure. The first speciation scenario was defined as allopatric speciation, characterized by complete geographic isolation without gene flow. The second speciation model accounted for post-divergence gene flow through isolation after migration (primary contact), while the third speciation model tested post-divergence with secondary contact, which led to recent gene flow between the two parasite populations. Finally, the fourth scenario hypothesized isolation with continuous gene flow between parasite populations (Fig. 4). See the supplementary Text S3, Supplementary Material online for a detailed explanation of the methodologies employed in this analysis.

### Isolation by Distance

To investigate a potential correlation between genetic distance and geographic distance among parasite populations, we analysed the Dataset B for an isolation by distance using the Mantel test in the adegenet package in R v.3.6.2 (Jombart 2008). This analysis required calculating the Nei genetic distances and the geographic distances (measured in kilometers between population locations), which were then correlated through the mantel.randtest function. To reduce the impact of host specificity on genetic distance, we extended the analysis to various genetic structures, reinforced by a Monte Carlo simulation with 999 permutations for stronger statistical inference. Following this, we sought to clarify the nature of the observed correlations, determining if they illustrated a continuous or a patchy distant cline of genetic differentiation. To do this, we employed a 2-dimensional Kernel density estimator through the kde2d function found in the MASS package in R, enhancing our grasp of the spatial distribution of genetic variation across the area.

### Gene Flow Among Parasite Populations

To investigate gene flow among sympatric host-parasite populations, we analysed SNP data from Czechia (Dataset D) using Treemix v.1.12 (Pickrell and Pritchard 2012) to create a maximum likelihood tree based on allelic frequencies, thereby identifying historical migration events. Furthermore, we assessed contemporary gene flow using Bayesian inference with BA3SNP software (Wilson and Rannala 2003; Mussmann et al. 2019), conducting a 10 million iteration analysis and discarding the initial 1 million as burn-in. Parameter adjustments were made for optimal acceptance rates, and CV was employed to ensure model accuracy. Detailed methodologies are provided in the supplementary text S4, Supplementary Material online.

### Genomic Signatures of Host-Specific Selection

To test for evidence of natural selection associated with host specificity, we analysed Dataset C to identify loci under selection between two distinct parasite populations in sympatric hosts. These populations exhibit marked host specificity, as evidenced by their genetic structure. To accurately identify loci under directional selection, we used multiple selection analyses and adopted a strategy that relies on the convergence of results from several analytical methods (Tsumura et al. 2012). We employed four well-established tests to detect outlier loci: the PCA-based method outlined in pcadapt v4.3.3 (Luu et al. 2017), the FST frequency method detailed in Outflank v2 (Lotterhos and Whitlock 2015), the Bayesian method that focuses on allele frequency variations as detailed in BayeScan v2.1 (Foll and Gaggiotti 2008), and the variance analysis in haplotype frequencies conducted using the hapFLK v1.4 (Fariello et al. 2013; detailed methodologies are in the supplementary text S5, Supplementary Material online).

To understand how genomic divergence relates to patterns of within-population diversity and selection, we estimated *F*_ST_, Tajima's *D*, per locus absolute divergence (dXY), and nucleotide diversity (π) using the R package PopGenome (Pfeifer et al. 2014). We then plotted both *F*_ST_ and dXY values against pi. We expect dXY to be significantly higher for outlier loci compared with neutral loci in a scenario of divergence with gene flow, assuming a sufficient amount of time has elapsed since the initial divergence (Cruickshank and Hahn 2014). Moreover, dXY is expected to elevate in highly differentiated regions, resulting in a positive correlation between F_ST_ and dXY. Furthermore, we employed the “KStest” function from the GSAR R package (Rahmatallah et al. 2017) to perform the Kolmogorov–Smirnov *D* test, comparing Tajima's *D* values between outlier and neutral loci. This helped ascertain if the distributions of Tajima's *D* values from putative outlier loci were statistically different from those of putative nonoutlier loci. A significant difference (*P* < 0.05) in distributions would imply that outlier loci have been subjected to distinct evolutionary pressures (i.e. selection, genetic drift, gene flow) compared with the rest of the genome.

### Differentially Expressed Genes Associated With Host Specificity

14 RNA-seq samples were used to compare transcriptome differences between two parasite populations detected by population genetic structure. Trimmomatic v0.33 (Bolger et al. 2014) was used to discard low-quality reads from all samples. Following trimming, paired-end reads were mapped to the *L. intestinalis* reference genome using STAR (Dobin et al. 2013). To eliminate reads that mapped to multiple loci and also to sort and convert the filtered SAM files to BAM format, we used Samtools v1.18 (Danecek et al. 2021). Furthermore, the mapped reads counted for genomic features using featureCounts v1.06 in the Subread package (Liao et al. 2014). To normalize the raw counts, we applied the Trimmed Mean of M values method. The edgeR (Robinson et al. 2010) was used to estimate differential expression of genes between parasite populations based on their host specificity. Additionally, we applied the criteria of an absolute log-fold change >1 or <−1 and a *P*-value < 0.05 to identify differentially expressed genes (DEGs). Finally, using the plotMDS function in edgeR, we plotted the first two principal components to evaluate the general similarities and differences among all the transcriptomes.

### Functional Annotation of Loci Under Selection and DEGs

We implemented a two-step strategy to annotate the selected loci. In the first step, we identified outliers in the *Ligula* reference genome and retrieved the relevant annotation details using the Integrative Genomics Viewer (Robinson et al. 2011). Next, for SNPs located in nongene regions of the *Ligula* genome, we took into account a 201 bp sequence—comprising 100 bp both upstream and downstream of the SNP from the reference genome sequences. Using OmicsBox v3, we performed a comprehensive functional annotation of these sequences (Gotz et al. 2008). Initially, nucleotide sequences (201 bp) were blasted to the NCBI nonredundant nucleotide (nt) database through BLASTX. For each search, we annotated the primary hit with the highest total score, maintaining an E-score of 10^−5^ for BLASTX and 10^−15^ for BLASTN, and insisting on a search coverage of more than 70%. We further enriched our annotations using the integrated InterProScan v5 module for domain-based information and mapped our data to the EggNOG database v6 to provide broader functional insights.

GO annotations for *L. intestinalis* genes were sourced from Nazarizadeh et al. (2024). We conducted functional enrichment analysis using the GSEA function in the clusterprofiler R package v4 (Wu et al. 2021), applying the Benjamini-Hochberg false discovery rate adjustment with a 0.05 threshold. We used the DOSE R package to create dot plots and enrichment maps for the highlighted genes. GOs were summarized via REVIGO (revigo.irb.hr) for DEG sets, adopting a 0.4 threshold and using SimRel for similarity.

## Supplementary Material

msaf163_Supplementary_Data

## Data Availability

All ddRAD and transcriptome data used in this study are available in the National Center for Biotechnology Information (NCBI) database under BioProject number PRJNA1088389 (https://www.ncbi.nlm.nih.gov/bioproject/PRJNA1088389). Additionally, all VCF files generated from genotype calling and simulation scripts used in the fastsimcoal analysis are publicly available on Mendeley Data (DOI: 10.17632/z85pg48f4h.2).
