# Supplementary material for: Host-Associated Genetic Differentiation in the Face of Ongoing Gene Flow: Ecological Speciation in a Pathogenic Parasite of Freshwater Fish
Source: Mol Biol Evol. 2025 Jul 8;42(7):msaf163. doi: 10.1093/molbev/msaf163 (PMC12301072; doi:10.1093/molbev/msaf163)
Supplement: msaf163_Supplementary_Data [file msaf163_supplementary_data.zip › Final_supporting_information.pdf]

Supplementary Materials for

Host-associated genetic differentiation in the face of ongoing gene flow: ecological speciation in a pathogenic parasite of freshwater fish

Masoud Nazarizadeh[1,2], Milena Nováková[2], Jakub Vlček[1,2], Jan Štefka[1,2*]

[1] Faculty of Science, University of South Bohemia in České Budějovice, Branišovská 1760, 37005 České Budějovice, Czechia

[2] Institute of Parasitology, Biology Centre, CAS, České Budějovice, Branišovská 31, 37005 České Budějovice, Czechia

[*]corresponding author: jan.stefka@prf.jcu.cz, tel: +420387775409, Faculty of Science, University of South Bohemia in České Budějovice, Branišovská 1760, 37005 České Budějovice, Czechia

This pdf file includes:

Supplementary text S1 to S5
Figs. S1 to S9
Tables S1 and S2
References

Supplementary text S1: ddRAD data assembly and SNP calling

Using the process_radtags program from Stacks v.2.5.6 (Rochette et al. 2019), we removed cut sites, barcodes, and adaptors from the ddRAD results, an essential step to ensure data integrity. Subsequently, we assessed the initial quality of our raw sequencing data using FastQC (Andrews 2010) and MultiQC (Ewels et al. 2016), which provide comprehensive quality control reports. For genotype calling and locus assembly in Stacks, we implemented specific criteria to ensure robust data analysis: we included loci present across all populations (-p option) and in at least 80% of individuals within each population (-r option), set a minor allele frequency threshold of 3% (--min-mac), and imposed a maximum observed heterozygosity limit of 80%. In our subsequent analyses focused on population genetic structure and FST-based evaluations, we excluded SNPs in linkage disequilibrium (LD) using the --write-random-snp option in Stacks' population program. To further refine our dataset, we employed vcftools v0.1.16 (Danecek et al. 2011) during post-processing to remove variants with extremely low (<5x) or high (>800x) coverage depth and loci with more than 20% missingness.

Supplementary text S2: Population genetic structure

To investigate the impact of host specificity on the genetic structure of parasite populations, we conducted genetic clustering analyses on two datasets: all samples in Lineage A (Dataset B) and only samples from Czechia (Dataset D), aiming to compare the genetic structure of parasite populations on both large and local scales. Firstly, we performed a discriminant analysis of principal components (DAPC) utilising the adegenet package (Jombart 2008; Jombart et al. 2010) in R v4.0.5. Using the K-means method, we determined the optimal number of genetic clusters based on the lowest Bayesian Information Criterion (BIC) value. Subsequently, individuals were assigned to clusters using DAPC and the optim.a.score method to establish the number of principal components to retain. We then visualised the individual

memberships in each cluster through plots and showcased the clusters using Principal Component Analysis (PCA).

Secondly, we employed the evolutionary clustering method in ADMIXTURE (Alexander et al. 2009), leveraging the parallel processing capabilities of AdmixPiPe (Mussmann et al. 2020). In this step, we conducted 20 replicates for each K value ranging from 1 to 7. The best K values were determined by the lowest cross-validation error (CV) across replicates, as suggested by Alexander and Lange (2011). The clustering was displayed using the CLUMPAK server (http://clumpak.tau.ac.il/).

Lastly, we used fineRADstructure and RADpainter v.0.2 (Malinsky et al. 2018) to explore the population genetic structure based on the nearest-neighbour haplotype. We ran the Stacks2fineRAD.py script from the fineRADstructure package to calculate the distribution of alleles and SNPs per locus, and the amount of missing data per individual, allowing a maximum of 10 SNPs per locus and limiting individual missingness to 25% during the conversion of the haplotype file to the RADpainter format. Considering the sensitivity of ddRAD to batch effects caused by minor differences between libraries at the size selection step, we examined the potential impact of missing data on any library-based structure. We utilised the fineRADstructure pipeline with default settings but increased the burn-in iterations to 200,000, with 1,000,000 iterations sampled at 1000 intervals. We evaluated convergence by assigning individuals to populations across multiple independent runs, reviewing the plots for the MCMC output of parameter values to ensure consistent convergence on Bayesian posterior distributions, and obtaining effective parameter sample sizes by lengthening the duration of each chain. To plot the co-ancestry heatmap, we utilised the “FinestructureLibrary.R” function in the fineRADstructure package (Malinsky et al. 2018).

Supplementary text S3: Coalescent analysis of speciation modelling

We calculated the composite likelihood of our observed data within a particular model using the site frequency spectrum (SFS) and the simulation method provided by fastsimcoal2 v2.7.9.3 (Excoffier and Foll 2011; Excoffier et al. 2021). For the simulations, we selected 25 samples for each genetic group representing the highest probability of assignment to their specific genetic cluster (i.e., we excluded highly admixed parasite populations between two genetic clusters; see the Results section). In the genotype calling process for our 50 samples, we utilised the Stacks software, adopting a stringent selection criterion to substantially reduce the incidence of missing data. This approach involved retaining only those loci that were consistently present across all individuals in both populations (r=1 and p=2). After applying these rigorous criteria, the genotype calling process yielded 30,442 SNPs. Then, using the easySFS Python script (https://github.com/isaacovercast/easySFS) , we generated a folded joint SFS, considering a single SNP for each locus to reduce the effects of linkage disequilibrium. The average mutation rate per site per generation (2.89×10^{-9}) and the divergence time events were obtained from (Nazarizadeh et al. 2023)Nazarizadeh *et al. (27)* (see Figure S1). The divergence time between two parasite lineages was estimated at 300 Kyr (Nazarizadeh et al. 2023; 95% highest posterior density interval: 160-457 Kyr). Given the lack of a precise divergence time between the two parasite lineages, we conducted the analysis at the lower bound splits. The time of change in demographic events was estimated as a model parameter and allowed to range from 1 to 160 Kyr (generations).

For each demographic model, we utilized fastsimcoal to optimize the fit to the observed multidimensional minor allele SFS via the composite-likelihood method. We employed specific options, starting with -N 100,000 to denote the number of coalescent simulations, followed by -C 10, which sets the threshold for observed SFS entry count by pooling all entries

with fewer than 10 SNPs. Subsequently, we applied -L 40, maintaining the number of expectation-maximization (EM) cycles, and -M 0.001 to define the minimum relative difference in parameter values for the stopping criterion. We assigned wide search ranges with log-uniform distributions to all model parameters (Excoffier and Foll 2011). To ascertain the parameter estimates that yield the highest likelihood, we executed 500 independent runs of fastsimcoal for each model (Excoffier et al. 2021).We used an information-theoretic model selection approach based on the Akaike's information criterion (AIC) to determine the probability of each model given the observed data. AIC scores were calculated using the calculateAIC.sh script (Meier et al. 2017) . AIC values for each model were rescaled (DAIC) by calculating the difference between the AIC value of each model and the minimum AIC obtained among all competing models. We also calculated Akaike weights (AIC_w) from these ΔAIC values using the standard likelihood-based formula (Guthery et al. 2003), which reflects the relative probability of each model being the best among those tested. Log-likelihood values are reported in base-10 (log10) scale for consistency with fastsimcoal2 outputs, as shown in Table TS2. Point estimates of the different demographic parameters for the best-supported model were selected from the run with the highest maximum composite likelihood. Finally, we calculated confidence intervals of parameter estimates from 100 parametric bootstrap replicates by simulating SFS from the maximum composite likelihood estimates and re-estimating parameters each time (Excoffier and Foll 2011; Excoffier et al. 2013).

Supplementary text S4: Gene flow among parasite populations

To evaluate gene flow among parasite populations from sympatrically occuring hosts, we analysed unlinked single nucleotide polymorphism (SNP) data from Czechia (Dataset D) to reconstruct interactions among the populations. We employed the Treemix v.1.12 tool (Pickrell and Pritchard 2012) to investigate gene flow among populations within a phylogenetic

framework. A Maximum Likelihood (ML) tree, based on allelic frequency data, was constructed to infer historical migration events between populations. We calculated individual migration events (n) separately. The second double-digest restriction-site associated DNA (ddRAD) dataset was categorized into six clusters according to host specificity, utilizing data from five distinct host-derived parasite populations. We assessed a series of migration events (m), ranging from 1 to 6 (1+ the total number of populations). The optimal model was selected based on the covariance associated with each migration event, and the tree's stability was confirmed through bootstrap analysis using 1,000 SNP blocks. Results from Treemix were visualized using the popcorn package in R.

Supplementary text S5: Genomic signatures of host specific selection

Furthermore, we analysed the contemporary gene flow among parasite groups from different hosts using Bayesian inference, facilitated by the BA3SNP software (Mussmann et al. 2019), which is specifically designed for this type of genetic analysis. The Bayesian framework allowed us to probabilistically estimate gene flow parameters, incorporating uncertainty in our models. Our analysis involved 10 million iterations, with the initial 1 million steps discarded as burn-in to ensure convergence and stability of the results. Data were sampled at every 1,000th step. To enhance the reliability of our findings, we performed cross-validation on the SNP datasets, which helps in assessing the model's predictive accuracy. We adjusted the -a (allele frequencies) and -f (inbreeding coefficients) parameters within the software to achieve optimal acceptance rates of 20 to 60%, respectively (*36*).

The first outlier detection method, utilised via the R package pcadapt v4.3.3 (Luu et al. 2017), conducts an individual-based genome scan grounded in PCA. This method does not rely on prior assumptions about population groupings, thus avoiding the need to force admixed individuals into predefined populations. The initial pcadapt run was performed using 20 PCs

(K=20). The ideal number of PCs to retain for later tests was determined following Cattell's rule, as described by Luu et al. (2017). The second outlier method used the Outflank r package (Whitlock and Lotterhos 2015) to determine the distribution of FST for neutral loci, which was then used to assign q-values to each locus to detect outliers that may be due to spatially heterogeneous selection. In this analysis, we set the 'number_of_samples' parameter to 5 (equal to the number of populations sampled), the 'LeftTrimFraction' to 0.08, the 'RightTrimFraction' to 0.30, and maintained the default setting for the Hmin parameter (0.1). The initial threshold for calculating q-values was 0.05, as set by default (Whitlock and Lotterhos 2015).

Additionally, we utilised the BayeScan v2.1 (Foll and Gaggiotti 2008) to pinpoint loci undergoing divergent selection, a process grounded in the variations in allele frequencies across distinct populations. SNP loci with a false discovery rate (FDR) below 0.05 were chosen as outlier SNPs. The analysis included an initial 20 pilot runs, each with 5000 iterations, succeeded by a main phase of 500,000 iterations with a burn-in period of 250,000 steps to guarantee convergence. The default prior odds value of 10 was retained throughout. Based on their alpha values, the loci were categorized: loci significantly above zero were regarded as under directional selection, while those below zero were considered to be under balancing selection (Foll and Gaggiotti 2008; Moore et al. 2014), with the rest being categorized as neutral. The resulting data, which included F_{ST} values, were imported into R software using the BayeScan package for further analysis following Geweke's diagnostic method, known for its efficient convergence diagnostics and outlier detection. This yielded a comprehensive list and tally of outliers, aiding in the visualization and interpretation of selection patterns across the examined populations.

Furthermore, the HapFLK software (Fariello et al. 2013) was employed for a haplotype-based analysis on regions potentially experiencing selection, with the local haplotype cluster (K) set

to 20 and the number of iterations increased to 20, balancing accuracy and computational time. Essentially, HapFLK expands on FST-based analysis, pinpointing genomic areas with notable haplotype divergence between individuals from selected populations, while acknowledging the population structure. This method is efficient in identifying recent selective sweeps differentiating the populations under study (Fariello et al. 2013). Moreover, regions were identified as potential selection areas if at least two successive SNPs showed a nominal p-value less than or equal to 0.05. To ascertain the random appearance of regions fulfilling this criterion, a resampling test was conducted, where SNP positions were shuffled 1000 times to determine the probability of observing sequences with a specific number of consecutive SNPs meeting the defined criteria. Regions with a resampling p-value under 0.05 were retained for additional analysis. Loci identified as outliers by the four methods were deemed "potential outliers" and were presented through Venn diagrams utilizing the "VennDiagram" package in R.

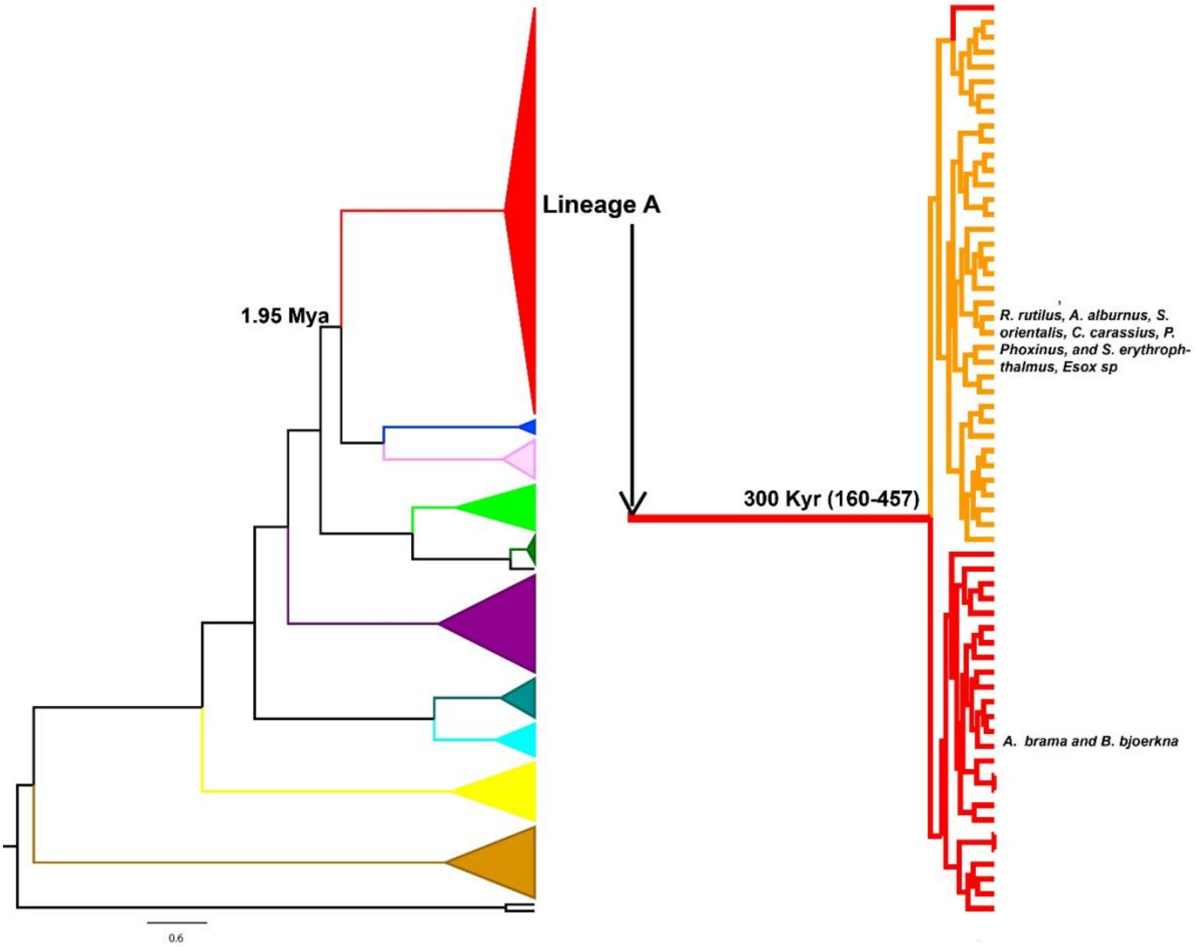

Fig. S1. Dated phylogenetic tree derived from ddRAD sequence data, adapted from Nazarizadeh et al. 2023. The tree illustrates the evolutionary relationships among parasite populations. Lineage A, depicted in red, indicates two distinct sub-lineages: one found in *Abramis brama* and *Blicca bjoerkna*, and the other in *Rutilus rutilus, Scardinius erythrophthalmus, S. orientalis, Alburnus alburnus, C. Carassius, P. Phoxinus, and Esox sp* . These sub-lineages diverged approximately 300 thousand years ago (Kyr), with the base of Lineage A tracing back to 1.95 million years ago (Mya).

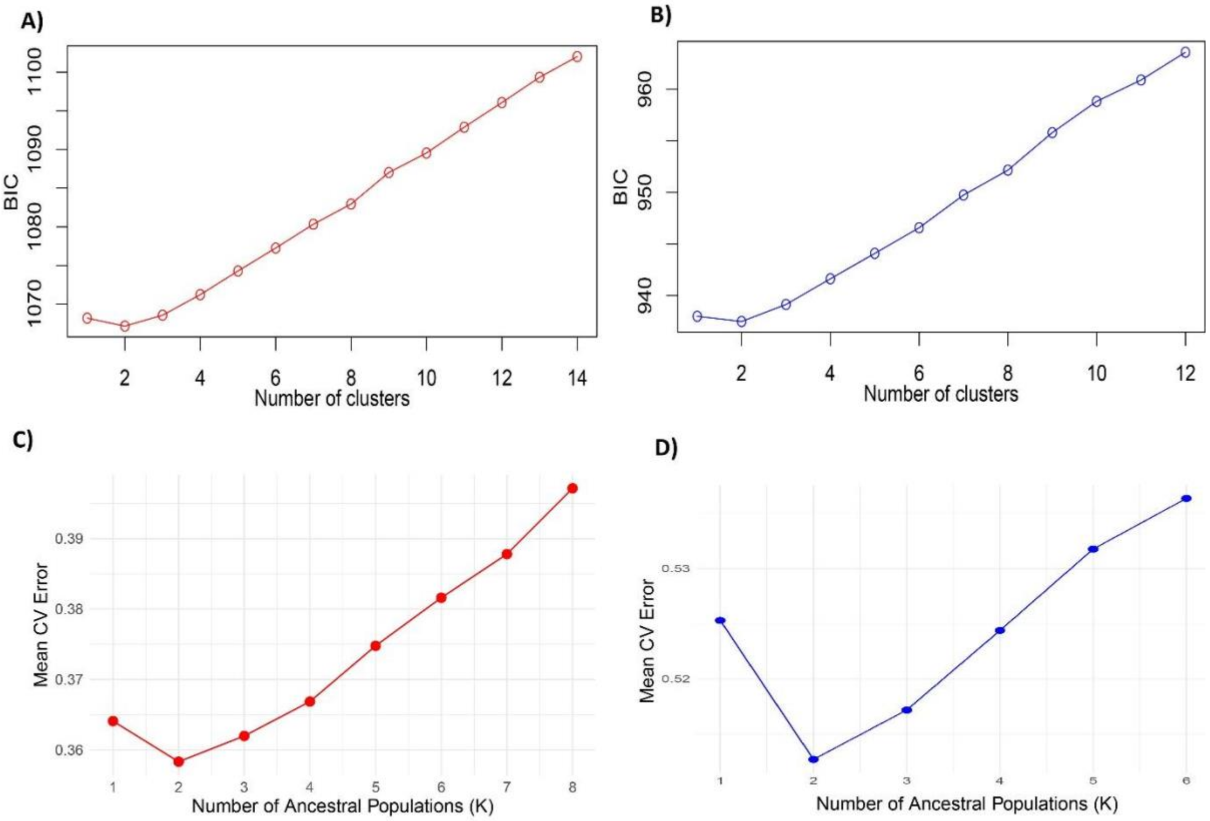

Fig. S2. Comparison of genetic clustering Analysis using DAPC and Admixture for Datasets B and D. A) Bayesian Information Criterion (BIC) scores from DAPC for Dataset B indicate an elbow shape for two genetic clusters. B) BIC scores similarly from DAPC for Dataset D also support the existence of two clusters. C) Mean Cross-validation (CV) error from Admixture analysis for Dataset B minimizes at two ancestral populations, indicating the most probable number of genetic clusters in Lineage A across all geographic locations. D) Mean CV error from Admixture analysis for Dataset D shows the lowest point at two ancestral populations, suggesting the optimal number of genetic clusters specific to Czech populations.

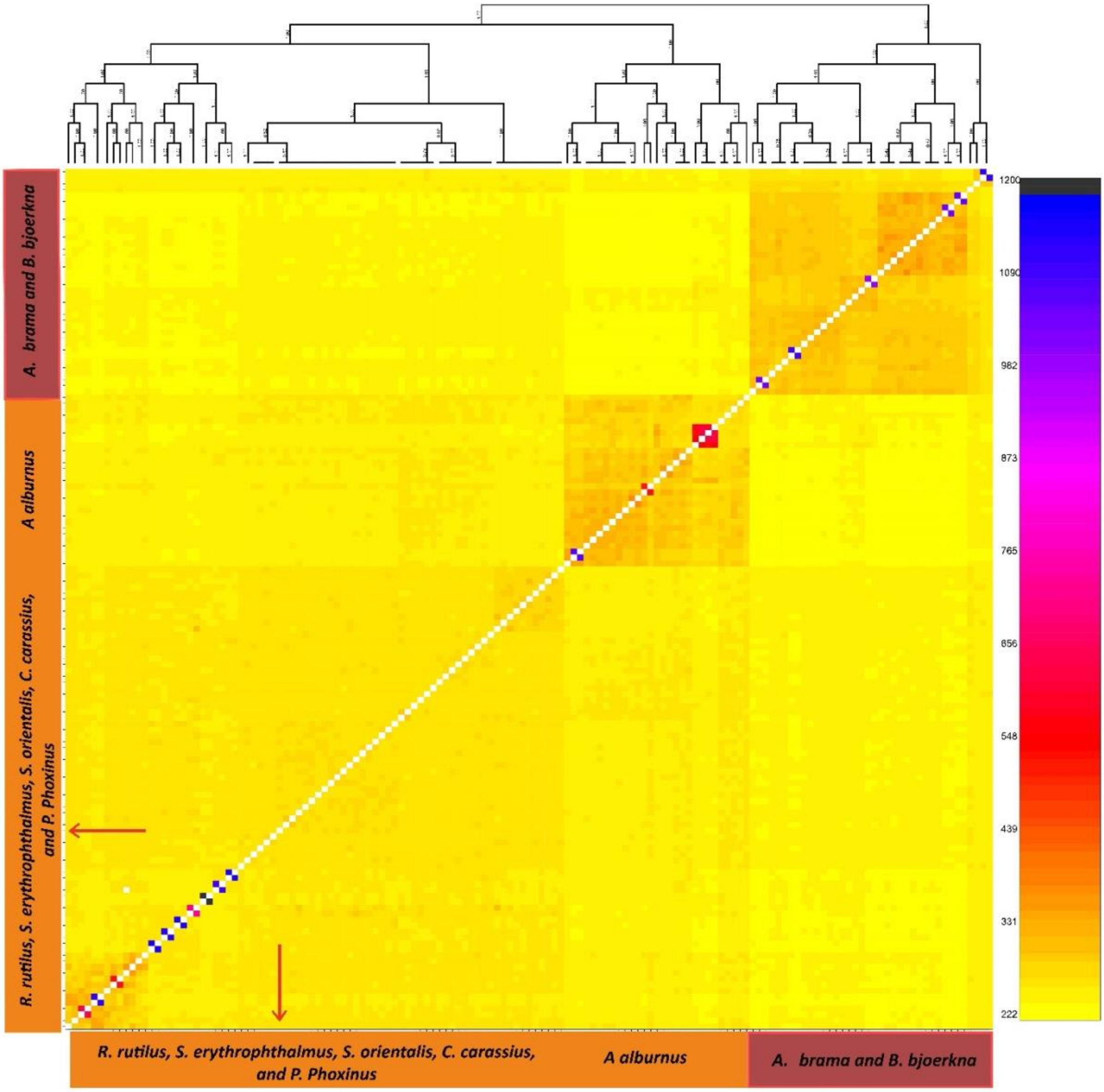

Fig. S3. Heatmap from FineRADstructure analysis illustrating genetic differentiation within Lineage A of the parasite *L. intestinalis*, revealing two distinct genetic clusters associated with different host species. The first cluster is specific to *A. brama* and *B. bjoerkna* (top left and bottom right), while the second cluster (bottom left) includes a broader range of hosts: *R. rutilus*, *A. alburnus*, *S. erythrophthalmus*, *S. orientalis*, *C. carassius*, and *P. phoxinus*.

Red arrows indicate a single *B. bjoerkna* sample clustering within the second group, suggesting a host switch and a close genetic relationship between the clusters.

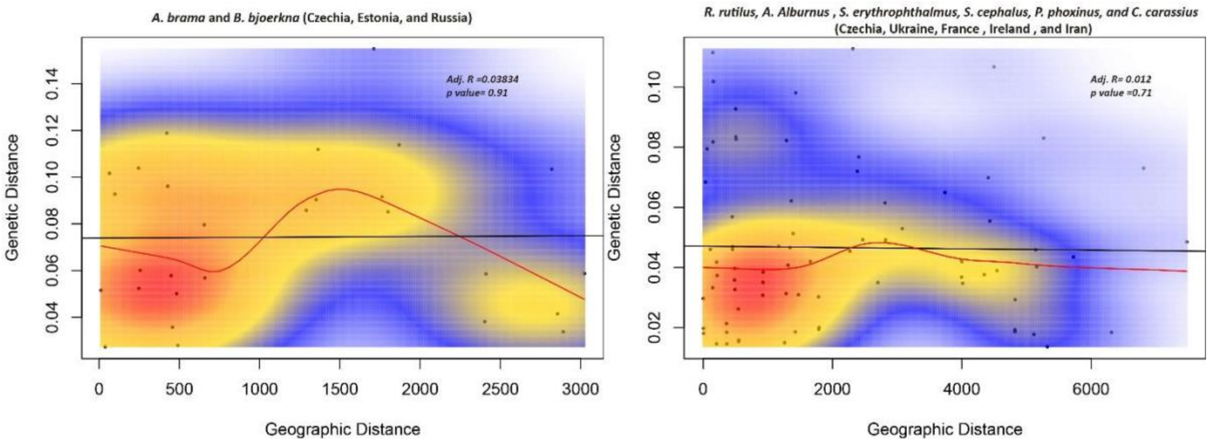

Fig. S4. Comparative Mantel tests showing no significant correlation between genetic and geographic distances in two parasite genetic clusters. The left panel represents a cluster comprising *A. brama* and *B. bjoerkna* populations from Czechia, Estonia, and Russia, while the right panel includes *R. rutilus*, *S. erythrophthalmus*, *A. alburnus*, *P. phoxinus*, and *C. carassius* populations from Czechia, Ukraine, France, Ireland, and Iran. Both analyses yield low and nonsignificant adjusted R-values, indicating that geographic separation does not explain genetic differentiation within these clusters.

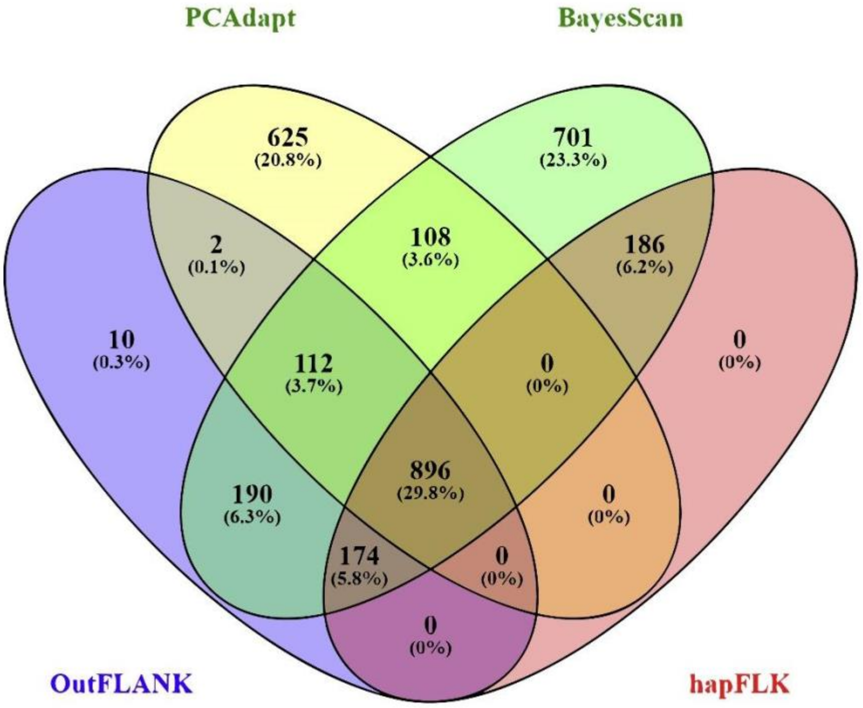

Fig. S5. Venn diagram depicting the overlap of genome-wide SNP outlier analyses using four different selection analysis methods: PCAadapt, BayeScan, OutFLANK, and hapFLK. The intersection at the center represents 896 SNPs (29.8% of the total) identified as under selection by all methods, highlighting a consensus in the detection of selective pressures across the genome.

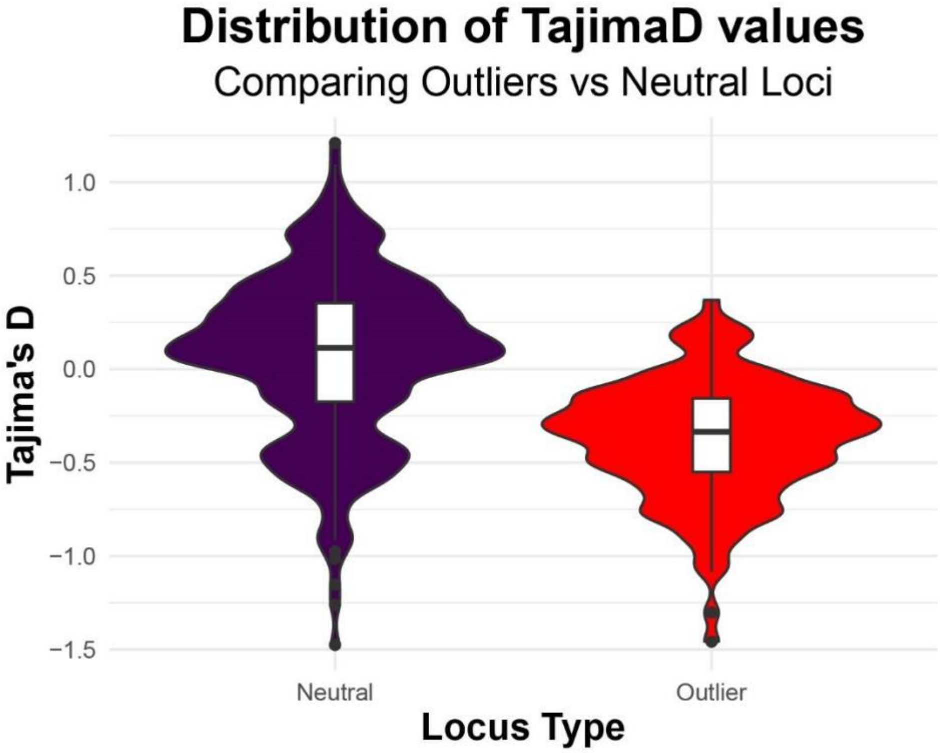

Fig. S6. Violin plot contrasting the distribution of Tajima's D values between neutral loci (purple) and outlier loci (red). Outlier loci show a significantly negative distribution of Tajima's D values, suggesting a deviation from neutrality, potentially due to selective sweeps.

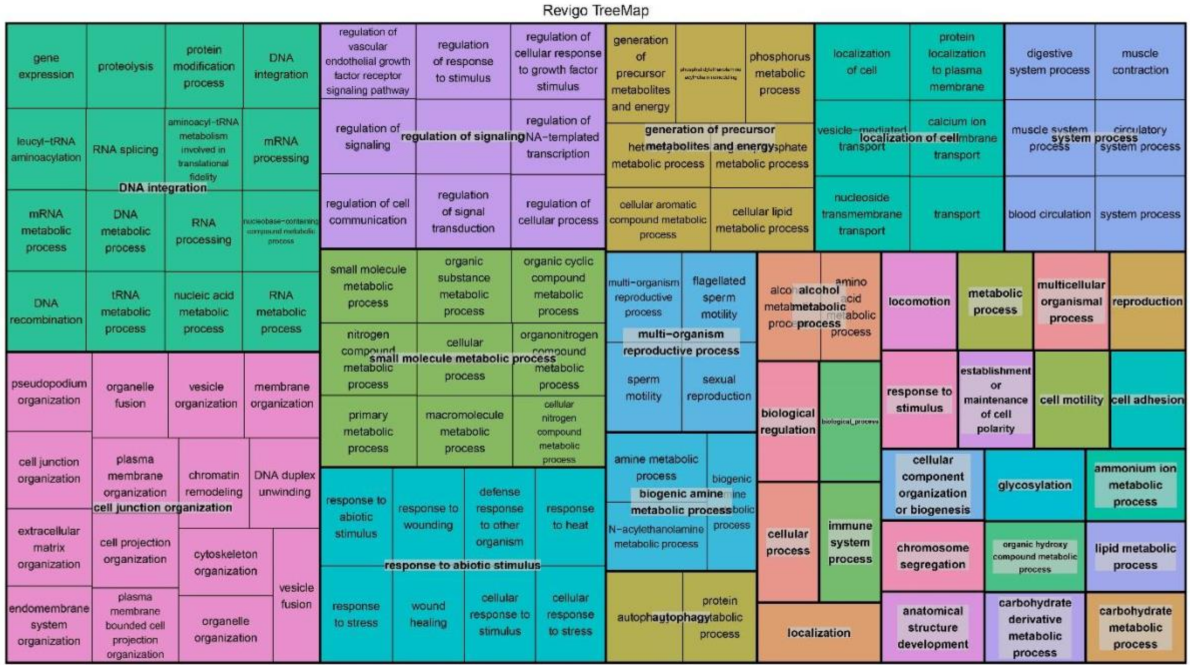

Fig. S7. Revigo TreeMap visualization of the functional annotation of SNPs under selection, categorized by biological processes. Each colored block represents a unique cluster

of related biological functions, with size indicating the frequency of the process in the dataset. This map highlights the diverse biological processes potentially influenced by selective pressures.

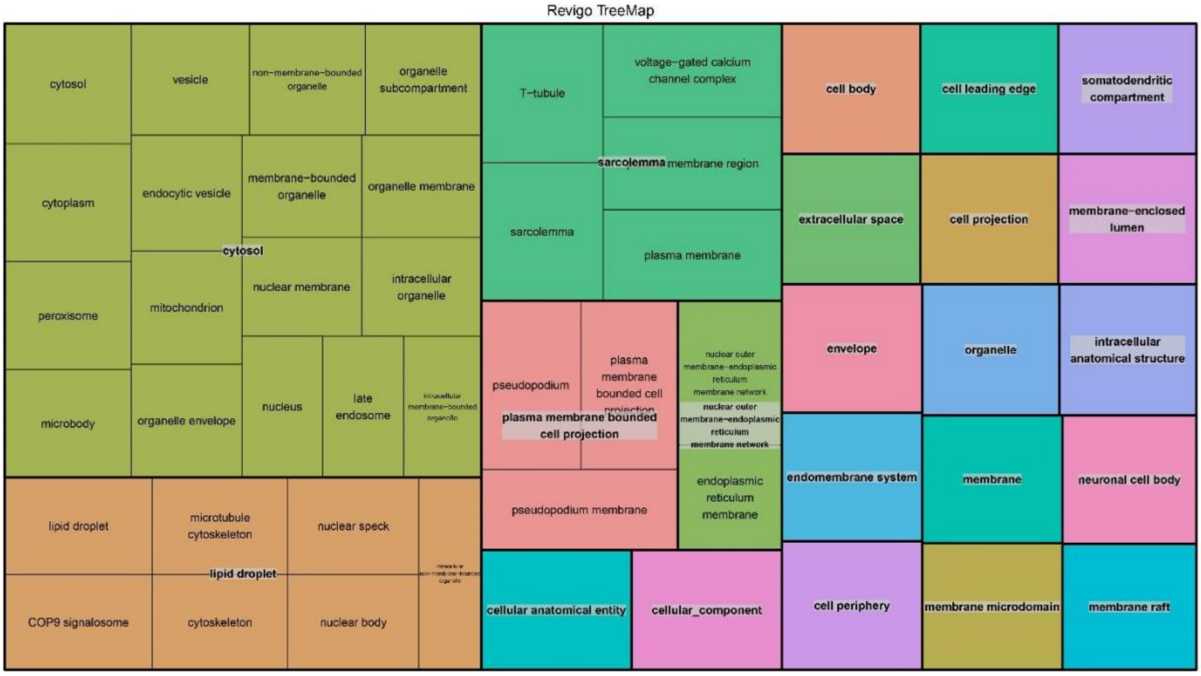

Fig. S8. Revigo TreeMap visualization representing the cellular component categorization of SNPs under selection. Each block signifies a distinct cellular component, with size corresponding to the prevalence of SNPs associated with that component in the data. This distribution showcases the complexity of cellular architecture impacted by selection, ranging from broader components like the cytosol and plasma membrane to specific structures such as lipid droplets and organelle membranes

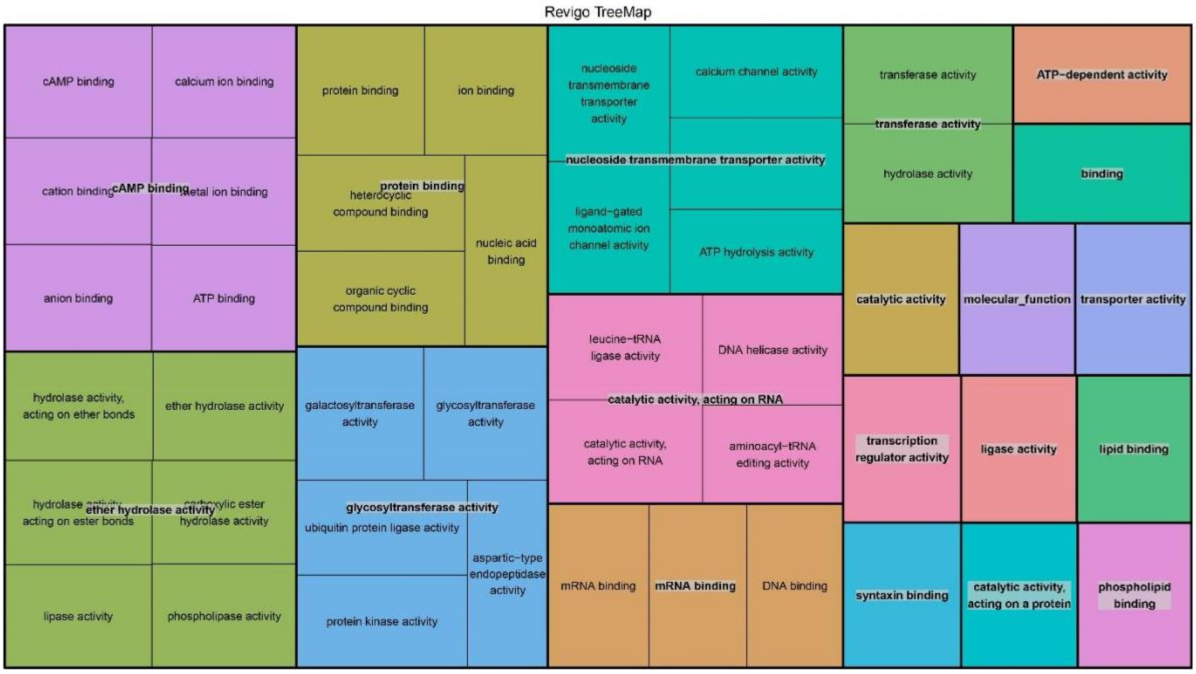

Fig. S9. Revigo TreeMap visualization mapping the molecular function categorization of SNPs under selection. Each block represents a distinct molecular function, with size proportional to the occurrence of SNPs associated with that function in the dataset. The map illustrates the intricate network of molecular activities, from binding and catalytic functions to transport and enzyme activities, influenced by selective pressures at the molecular level.

Table S1. List of DNA and RNA samples and their Genbank accession numbers.

DNA sample ID	Host	Locality	Country	Number of ddRAD reads	Barcode	Index	Accession number	Reference
AB1	*Blicca bjoerkna*	Římov	Czechia	5028862	CTGAT	ATCACG	SAMN32032486	Nazarizadeh *et al.* (*27*)
AB2	*Blicca bjoerkna*	Římov	Czechia	4445081	CTGCG	ATCACG	SAMN32032487	
AbLipno1	*Abramis brama*	Lipno	Czechia	5418712	GAGAT	ATCACG	SAMN32032488	
AbLipno2	*Abramis brama*	Lipno	Czechia	5094581	GAGTC	ATCACG	SAMN32032489	
Li12_L1	*Abramis brama*	Lipno	Czechia	4818508	GGTTG	ATCACG	SAMN32032371	
Li14	*Abramis brama*	Lipno	Czechia	5286573	CGTAC	ATCACG	SAMN32032484	

Li9_L1	*Abramis brama*	Lipno	Czechia	5571409	TGCAT	ATCACG	SAMN32032370	
LIPC1	*Abramis brama*	Lipno	Czechia	6191387	CGGTA	CGATGT	SAMN32032444	
X16Ab	*Abramis brama*	Římov	Czechia	3375762	ACGGT	ATCACG	SAMN32032474	
X2Ab	*Abramis brama*	Římov	Czechia	3418401	AAGGA	ATCACG	SAMN32032470	
X3Ab	*Abramis brama*	Římov	Czechia	4630225	AGCTA	ATCACG	SAMN32032471	
X4Ab	*Abramis brama*	Římov	Czechia	3987628	ACACA	ATCACG	SAMN32032472	
CE2_L3	*Blicca bjoerkna*	Medard	Czechia	10733223	TCACG	CGATGT	SAMN32032398	
CF_L1	*Blicca bjoerkna*	Medard	Czechia	6221424	CGGCT	ATCACG	SAMN32032373	
Li2	*Blicca bjoerkna*	Lipno	Czechia	5036084	CGGTA	ATCACG	SAMN32032483	
Li3_L1	*Blicca bjoerkna*	Lipno	Czechia	5486672	CGATC	ATCACG	SAMN32032368	
Li5_L1	*Blicca bjoerkna*	Lipno	Czechia	5576052	TCGAT	ATCACG	SAMN32032369	
MPL27B	*Rutilus rutilus*	Most	Czechia	5807870	TTACC	ATCACG	SAMN32032502	
MPL28	*Rutilus rutilus*	Most	Czechia	6509615	CGATC	ATCACG	SAMN32032465	
MPL29	*Rutilus rutilus*	Most	Czechia	5019649	CAACC	ATCACG	SAMN32032468	
MPL30	*Rutilus rutilus*	Most	Czechia	4529673	GGTTG	ATCACG	SAMN32032469	
PA1_L1	*Rutilus rutilus*	Medard	Czechia	4733833	CGGTA	ATCACG	SAMN32032374	
PE1_L1	*Rutilus rutilus*	Medard	Czechia	6591323	CGTAC	ATCACG	SAMN32032375	
PK_L3	*Rutilus rutilus*	Medard	Czechia	9863929	TCCGG	CGATGT	SAMN32032399	
X20M11	*Rutilus rutilus*	Most	Czechia	5084222	GCCGT	ATCACG	SAMN32032490	
X20M34	*Rutilus rutilus*	Most	Czechia	5876404	GCTGA	ATCACG	SAMN32032491	
X20M48	*Rutilus rutilus*	Most	Czechia	6035246	CATAT	CGATGT	SAMN32032441	
X20M51	*Rutilus rutilus*	Most	Czechia	4112140	GTAGT	ATCACG	SAMN32032492	
X20R1PL	*Rutilus rutilus*	Římov	Czechia	5850754	CGTCG	ATCACG	SAMN32032485	

X9Rr	*Rutilus rutilus*	Římov	Czechia	4100447	AATTA	ATCACG	SAMN32032473	
MPR1	*Scardinius erythrophthalmus*	Most	Czechia	6283789	TCAGT	ATCACG	SAMN32032498	
MPR2	*Scardinius erythrophthalmus*	Most	Czechia	5779666	TCCGG	ATCACG	SAMN32032499	
MPR3	*Scardinius erythrophthalmus*	Most	Czechia	5855323	TCTGC	ATCACG	SAMN32032500	
X20M57	*Scardinius erythrophthalmus*	Most	Czechia	6310454	CTGAT	CGATGT	SAMN32032447	
X20M58	*Scardinius erythrophthalmus*	Most	Czechia	5502583	GTCCG	ATCACG	SAMN32032493	
E2b_L4	*Abramis brama*	Peipsi	Estonia	5157082	CATAT	TTAGGC	SAMN32032416	
Ea2	*Abramis brama*	Peipsi	Estonia	4480806	TAGTA	ATCACG	SAMN32032496	
TS04.126a_L4	*Abramis brama*	Rybinsk	Russia	4826446	CGGCT	TTAGGC	SAMN32032418	
TS04.126c_L4	*Abramis brama*	Rybinsk	Russia	6194506	CGTAC	TTAGGC	SAMN32032420	
TS04.126f_L4	*Abramis brama*	Rybinsk	Russia	4163635	CGTCG	TTAGGC	SAMN32032421	
TS04.126h_L4	*Abramis brama*	Rybinsk	Russia	5453257	CTGCG	TTAGGC	SAMN32032423	
UA1_L4	*Alburnus alburnus*	Dniester	Ukraine	6421321	GTAGT	TTAGGC	SAMN32032433	
UA2_L4	*Carassius carassius*	Dniester	Ukraine	5673661	GTCCG	TTAGGC	SAMN32032434	
S2_L1	*Phoxinus phoxinus*	Medard	Czechia	6082925	CGAAT	ATCACG	SAMN32032372	
CR22_L4	*Rutilus rutilus*	Créteil	France	7761401	TACCG	TTAGGC	SAMN32032436	
CR23_L4	*Rutilus rutilus*	Créteil	France	6568657	TACGT	TTAGGC	SAMN32032437	
CR24_L4	*Rutilus rutilus*	Créteil	France	6807948	TAGTA	TTAGGC	SAMN32032438	
CR25_L4	*Rutilus rutilus*	Créteil	France	6941629	TATAC	TTAGGC	SAMN32032439	

CR26_L4	*Rutilus rutilus*	Créteil	France	6179618	TCACG	TTAGGC	SAMN32032440	
FR75_L4	*Rutilus rutilus*	Créteil	France	4532929	GCATG	ATCACG	SAMN32032463	
FR77	*Rutilus rutilus*	Créteil	France	6005983	AACCA	ATCACG	SAMN32032464	
FR84	*Rutilus rutilus*	Most	Czechia	6509615	CGATC	ATCACG	SAMN32032465	
FR88	*Rutilus rutilus*	Créteil	France	5568505	TCGAT	ATCACG	SAMN32032466	
FR89	*Rutilus rutilus*	Créteil	France	4560249	TGCAT	ATCACG	SAMN32032467	
IE1_L4	*Rutilus rutilus*	Lough Neagh	Ireland	5816625	TCGAT	TTAGGC	SAMN32032403	
IE2_L4	*Rutilus rutilus*	Lough Neagh	Ireland	5905188	TGCAT	TTAGGC	SAMN32032404	
UA3_L4	*Rutilus rutilus*	Dniester	Ukraine	7927417	GTCGA	TTAGGC	SAMN32032435	
Ir8_L3	*Squalius orientalis*	Alborz Dam	Iran	8054152	GCTGA	CGATGT	SAMN32032506	
S5_2	*Squalius orientalis*	Alborz Dam	Iran	7117135	CTGTC	CGATGT	SAMN32032448	
S2	*Squalius orientalis*	Alborz Dam	Iran	6082925	CGAAT	ATCACG	SAMN32032372	
S6_1	*Squalius orientalis*	Alborz Dam	Iran	7005135	CTTGG	CGATGT	SAMN32032449	
S7_2	*Squalius orientalis*	Alborz Dam	Iran	6202440	GACAC	CGATGT	SAMN32032450	
1.1	*Rutilus rutilus*	Těrlicko	Czechia	14013944	CGAAT	ATCACG	SAMN40467638	Present study
1.2	*Rutilus rutilus*	Těrlicko	Czechia	14310996	CGGCT	ATCACG	SAMN40467639	
1.3	*Rutilus rutilus*	Těrlicko	Czechia	9511478	GGCCA	CGATGT	SAMN40467640	
1.4	*Rutilus rutilus*	Těrlicko	Czechia	14015584	CGGTA	ATCACG	SAMN40467641	
2.2	*Alburnus alburnus*	Těrlicko	Czechia	8205610	GGCTC	CGATGT	SAMN40467642	
2.3	*Alburnus alburnus*	Těrlicko	Czechia	16576476	CGTAC	ATCACG	SAMN40467643	
2.5	*Alburnus alburnus*	Těrlicko	Czechia	7792994	GTAGT	CGATGT	SAMN40467644	

2.5b	*Alburnus alburnus*	Těrlicko	Czechia	6347270	TCAGT	TTAGGC	SAMN40467645	
2.8	*Alburnus alburnus*	Těrlicko	Czechia	13205482	CGTCG	ATCACG	SAMN40467646	
3.1	*Abramis brama*	Těrlicko	Czechia	16011422	TTACC	ATCACG	SAMN40467647	
3.2	*Abramis brama*	Těrlicko	Czechia	14417060	TGGAA	ATCACG	SAMN40467648	
3.3	*Abramis brama*	Těrlicko	Czechia	8874996	GCATG	CGATGT	SAMN40467649	
3.4	*Abramis brama*	Těrlicko	Czechia	10308066	AACCA	CGATGT	SAMN40467650	
3.6	*Abramis brama*	Těrlicko	Czechia	9523808	CGATC	CGATGT	SAMN40467651	
3.7	*Abramis brama*	Těrlicko	Czechia	7721900	TCGAT	CGATGT	SAMN40467652	
5.1	*Rutilus rutilus*	Těrlicko	Czechia	5159322	TGCAT	CGATGT	SAMN40467653	
6.1	*Rutilus rutilus*	Těrlicko	Czechia	9069386	CAACC	CGATGT	SAMN40467654	
6.2	*Rutilus rutilus*	Těrlicko	Czechia	8919464	GGTTG	CGATGT	SAMN40467655	
6.3	*Rutilus rutilus*	Těrlicko	Czechia	8869364	AAGGA	CGATGT	SAMN40467656	
6.4	*Rutilus rutilus*	Těrlicko	Czechia	10515248	AGCTA	CGATGT	SAMN40467657	
7.1	*Rutilus rutilus*	Těrlicko	Czechia	10821072	ACACA	CGATGT	SAMN40467658	
8.2	*Abramis brama*	Těrlicko	Czechia	10160742	AATTA	CGATGT	SAMN40467659	
9.1	*Alburnus alburnus*	Žermanice	Czechia	13884178	CTGAT	ATCACG	SAMN40467660	
11.1	*Alburnus alburnus*	Žermanice	Czechia	8642264	GGATA	CGATGT	SAMN40467661	
13.1	*Alburnus alburnus*	Žermanice	Czechia	16636890	CTGCG	ATCACG	SAMN40467662	
14.1	*Abramis brama*	Žermanice	Czechia	15167330	CTGTC	ATCACG	SAMN40467663	
14.2	*Abramis brama*	Žermanice	Czechia	15642320	CTTGG	ATCACG	SAMN40467664	
20.1	*Alburnus alburnus*	Žermanice	Czechia	14217858	GACAC	ATCACG	SAMN40467665	
20.2	*Alburnus alburnus*	Žermanice	Czechia	13597458	GAGAT	ATCACG	SAMN40467666	
22.1	*Rutilus rutilus*	Žermanice	Czechia	9829370	ACGGT	CGATGT	SAMN40467667	

23.1	*Alburnus alburnus*	Žermanice	Czechia	7040012	CGGCT	CGATGT	SAMN40467668	
23.2	*Alburnus alburnus*	Žermanice	Czechia	7793932	ACTGG	CGATGT	SAMN40467669	
20LIPO19	*Alburnus alburnus*	Lipno	Czechia	6228372	GACAC	ATCACG	SAMN40467670	
20LIPO3	*Alburnus alburnus*	Lipno	Czechia	2888784	CTTGG	ATCACG	SAMN40467671	
20M40	*Rutilus rutilus*	Most	Czechia	6204690	GGATA	ATCACG	SAMN40467672	
20M44	*Rutilus rutilus*	Most	Czechia	5656754	GGCCA	ATCACG	SAMN40467673	
20M48	*Rutilus rutilus*	Most	Czechia	5983782	CATAT	CGATGT	SAMN40467674	
CNA	*Blicca bjoerkna*	Medard	Czechia	5343952	AATTA	ATCACG	SAMN40467675	
CND	*Blicca bjoerkna*	Medard	Czechia	5028252	ACGGT	ATCACG	SAMN40467676	
CZ77	*Alburnus alburnus*	Želivka	Czechia	8211794	GCATG	CGATGT	SAMN40467677	
CZ81	*Alburnus alburnus*	Želivka	Czechia	6836922	AACCA	CGATGT	SAMN40467678	
CZ82	*Alburnus alburnus*	Želivka	Czechia	7293922	CGATC	CGATGT	SAMN40467679	
CZ83	*Alburnus alburnus*	Želivka	Czechia	8166296	TCGAT	CGATGT	SAMN40467680	
CZ84	*Alburnus alburnus*	Želivka	Czechia	6212546	TGCAT	CGATGT	SAMN40467681	
CZ86	*Alburnus alburnus*	Želivka	Czechia	8275674	CAACC	CGATGT	SAMN40467682	
CZ90	*Rutilus rutilus*	Želivka	Czechia	6557476	GGTTG	CGATGT	SAMN40467683	
CZ92	*Rutilus rutilus*	Želivka	Czechia	7637546	AAGGA	CGATGT	SAMN40467684	
CZ94	*Rutilus rutilus*	Želivka	Czechia	5848190	AGCTA	CGATGT	SAMN40467685	
CZ95	*Rutilus rutilus*	Želivka	Czechia	7861020	ACACA	CGATGT	SAMN40467686	
CZ96	*Rutilus rutilus*	Želivka	Czechia	8651958	AATTA	CGATGT	SAMN40467687	
JO1	*Blicca bjoerkna*	Jordan	Czechia	5885294	ATGAG	ATCACG	SAMN40467688	
KL1	*Rutilus rutilus*	Klíčava	Czechia	5298614	GCATG	ATCACG	SAMN40467689	
KL1a	*Rutilus rutilus*	Klíčava	Czechia	10224808	GTCGA	CGATGT	SAMN40467690	

Li1	*Blicca bjoerkna*	Lipno	Czechia	10449928	TACCG	CGATGT	SAMN40467691	
Li1a	*Blicca bjoerkna*	Lipno	Czechia	5694296	AACCA	ATCACG	SAMN40467692	
Li11B	*Rutilus rutilus*	Lipno	Czechia	5773286	CAACC	ATCACG	SAMN40467693	
Li15	*Abramis brama*	Lipno	Czechia	5449370	AAGGA	ATCACG	SAMN40467694	
LIPC1	*Abramis brama*	Lipno	Czechia	5763502	CTGTC	ATCACG	SAMN40467695	
N1	*Rutilus rutilus*	Nýrsko	Czechia	3937244	ACGGT	CGATGT	SAMN40467696	
N2	*Rutilus rutilus*	Nýrsko	Czechia	6660272	ACTGG	CGATGT	SAMN40467697	
N3	*Rutilus rutilus*	Nýrsko	Czechia	5699488	ACTTC	CGATGT	SAMN40467698	
N4	*Rutilus rutilus*	Nýrsko	Czechia	5247764	ATACG	CGATGT	SAMN40467699	
N8	*Rutilus rutilus*	Nýrsko	Czechia	6743392	ATTAC	CGATGT	SAMN40467700	
NPL1	*Rutilus rutilus*	Nýrsko	Czechia	9241276	CTGAT	CGATGT	SAMN40467701	
NPL10A	*Rutilus rutilus*	Nýrsko	Czechia	10314500	GCCGT	CGATGT	SAMN40467702	
NPL2	*Rutilus rutilus*	Nýrsko	Czechia	10663280	CTGCG	CGATGT	SAMN40467703	
NPL3	*Rutilus rutilus*	Nýrsko	Czechia	10420452	CTGTC	CGATGT	SAMN40467704	
NPL4	*Rutilus rutilus*	Nýrsko	Czechia	10607450	CTTGG	CGATGT	SAMN40467705	
NPL6	*Rutilus rutilus*	Nýrsko	Czechia	8388230	GACAC	CGATGT	SAMN40467706	
NPL7	*Rutilus rutilus*	Nýrsko	Czechia	10834172	GAGAT	CGATGT	SAMN40467707	
NPL9	*Rutilus rutilus*	Nýrsko	Czechia	12124410	GAGTC	CGATGT	SAMN40467708	
NPL9b	*Rutilus rutilus*	Nýrsko	Czechia	8963938	TTACC	TTAGGC	SAMN40467709	
P10	*Rutilus rutilus*	Medard	Czechia	4259218	TCCGG	CGATGT	SAMN40467710	
P2	*Rutilus rutilus*	Medard	Czechia	5025966	TCAGT	CGATGT	SAMN40467711	
PB	*Rutilus rutilus*	Medard	Czechia	17189194	AGCTA	ATCACG	SAMN40467712	
PE	*Rutilus rutilus*	Medard	Czechia	4697584	ACTGG	ATCACG	SAMN40467713	

PH	*Rutilus rutilus*	Medard	Czechia	17671850	ACACA	ATCACG	SAMN40467714	
PK	*Rutilus rutilus*	Medard	Czechia	14748944	ACGGT	ATCACG	SAMN40467715	
PQ	*Rutilus rutilus*	Medard	Czechia	15839428	ACTGG	ATCACG	SAMN40467716	
PX	*Rutilus rutilus*	Medard	Czechia	6300810	ATACG	ATCACG	SAMN40467717	
ZE1	*Rutilus rutilus*	Želivka	Czechia	5976034	ATTAC	ATCACG	SAMN40467718	
ZE2	*Rutilus rutilus*	Želivka	Czechia	10892852	TAGTA	CGATGT	SAMN40467719	
ZE2a	*Rutilus rutilus*	Želivka	Czechia	6372262	CATAT	ATCACG	SAMN40467720	
**RNA sample ID**	**Host**	**Locality**	**Country**	**Number of Reads**	**GC %**	**Accession number**		
LB6	*Blicca bjoerkna*	Lipno	Czechia	75627444	47	SAMN40472618		
Li12	*Abramis brama*	Lipno	Czechia	61674254	46	SAMN40472620		
Li14	*Abramis brama*	Lipno	Czechia	75299606	46	SAMN40472621		
Li15	*Abramis brama*	Lipno	Czechia	69298210	46	SAMN40472622		
Li1	*Blicca bjoerkna*	Lipno	Czechia	64890334	48	SAMN40472619		
Li3	*Blicca bjoerkna*	Lipno	Czechia	73873790	47	SAMN40472624		
Li9	*Abramis brama*	Lipno	Czechia	61399578	46	SAMN40472625		Present study
Li	*Abramis brama*	Lipno	Czechia	89419634	47	SAMN40472623		
PA1	*Rutilus rutilus*	Medard	Czechia	49662844	49	SAMN40472626		
PK	*Rutilus rutilus*	Medard	Czechia	52638726	48	SAMN40472627		
PX	*Rutilus rutilus*	Medard	Czechia	53825102	49	SAMN40472628		
x20M40	*Rutilus rutilus*	Most	Czechia	50696116	48	SAMN40472629		
x20M44	*Rutilus rutilus*	Most	Czechia	79282210	47	SAMN40472630		
x20M51	*Rutilus rutilus*	Most	Czechia	64314292	47	SAMN40472631		

Table S2. Comparison of divergence scenarios for two parasite populations analyzed using fastsimcoal2." The populations are associated with *R. rutilus*, *S. erythrophthalmus*, and *A. alburnus* (RSA) hosts, and *A. brama* and *B. bjoerkna* (AB) hosts.

**Model Parameters**	**Search range (log uniform distribution)**	**Allopatry**	**primary contact**	**Secondary contact**	**Isolation with continuous gene flow**
**RSA**	100-100000	11,561	7,070	7,861	**5,211**
**AB**	100-100000	48,323	5,541	4,935	**5,332**
**MRCA (LineageA)**	resize 0.1-10	11,549	3,723	3,029	**3,627**
**Time**	1- 160,000	NA	72000	9,100	**58,000**
**early mig RSA->AB**	$1x10^{-6}$-0.01	NA	NA	$3.3x10^{-4}$/1.64	**$4.2x10^{-6}$/0.02**
**early mig AB->RSA**	$1x10^{-6}$-0.01	NA	NA	$6.8x10^{-4}$/4.00	**$3.3x10^{-4}$/1.96**
**recent mig RSA->AB**	$1x10^{-6}$-0.01	NA	$4.4x10^{-4}$/2.42	NA	**$9.4x10^{-4}$/5.00**
**recent mig AB->RSA**	$1x10^{-6}$-0.01	NA	$9.4x10^{-4}$/6.66	NA	**$7.1x10^{-4}$/4.22**
Log10		-701.5	-686.1	-686.7	-680.6
DAIC		96.4	25.4	28.1	**0.00**
AIC		4367.2	3821.6	3867.5	**3150.2**
AIC_w		0.00	0.19	0.14	**0.81**

References:

Alexander DH, Lange K. 2011. Enhancements to the ADMIXTURE algorithm for individual ancestry estimation. BMC Bioinformatics 12: 1–6.

Alexander DH, Novembre J, Lange K. 2009. Fast model-based estimation of ancestry in unrelated individuals. Genome Res. 19: 1655–1664.

Andrews S. 2010. FastQC: a quality control tool for high throughput sequence data.

Danecek P, Auton A, Abecasis G, Albers CA, Banks E, DePristo MA, Handsaker RE, Lunter G, Marth GT, Sherry ST. 2011. The variant call format and VCFtools. Bioinformatics 27: 2156–

2158.

Ewels P, Magnusson M, Lundin S, Käller M. 2016. MultiQC: summarize analysis results for multiple tools and samples in a single report. Bioinformatics 32: 3047–3048.

Excoffier L, Dupanloup I, Huerta-Sánchez E, Sousa VC, Foll M. 2013. Robust demographic inference from genomic and SNP data. PLoS Genet. 9: e1003905.

Excoffier L, Foll M. 2011. Fastsimcoal: a continuous-time coalescent simulator of genomic diversity under arbitrarily complex evolutionary scenarios. Bioinformatics 27: 1332–1334.

Excoffier L, Marchi N, Marques DA, Matthey-Doret R, Gouy A, Sousa VC. 2021. fastsimcoal2: demographic inference under complex evolutionary scenarios. Bioinformatics 37: 4882–4885.

Fariello MI, Boitard S, Naya H, SanCristobal M, Servin B. 2013. Detecting signatures of selection through haplotype differentiation among hierarchically structured populations. Genetics 193: 929–941.

Foll M, Gaggiotti O. 2008. A genome-scan method to identify selected loci appropriate for both dominant and codominant markers: a Bayesian perspective. Genetics 180: 977–993.

Guthery FS, Burnham KP, Anderson DR. 2003. Model selection and multimodel inference: a practical information-theoretic approach. J. Wildl. Manage. 67: 655.

Jombart T. 2008. adegenet: an R package for the multivariate analysis of genetic markers. Bioinformatics 24: 1403–1405.

Jombart T, Devillard S, Balloux F. 2010. Discriminant analysis of principal components: a new method for the analysis of genetically structured populations. BMC Genet. 11: 94.

Luu K, Bazin E, Blum MGB. 2017. pcadapt: an R package to perform genome scans for selection based on principal component analysis. Mol. Ecol. Resour. 17: 67–77.

Malinsky M, Trucchi E, Lawson DJ, Falush D. 2018. RADpainter and fineRADstructure: population inference from RADseq data. Mol. Biol. Evol. 35: 1284–1290.

Meier JI, Sousa VC, Marques DA, Selz OM, Wagner CE, Excoffier L, Seehausen O. 2017. Demographic modelling with whole-genome data reveals parallel origin of similar Pundamilia cichlid species after hybridization. Mol. Ecol. 26: 123–141.

Moore J, Bourret V, Dionne M, Bradbury I, O'Reilly P, Kent M, Chaput G, Bernatchez L. 2014. Conservation genomics of anadromous Atlantic salmon across its North American range: outlier loci identify the same patterns of population structure as neutral loci. Mol. Ecol. 23: 5680–5697.

Mussmann SM, Douglas MR, Chafin TK, Douglas ME. 2019. BA3-SNPs: contemporary migration reconfigured in BayesAss for next-generation sequence data. Methods Ecol. Evol. 10: 1808–1813.

Mussmann SM, Douglas MR, Chafin TK, Douglas ME. 2020. AdmixPipe: population analyses in Admixture for non-model organisms. BMC Bioinformatics 21: 337.

Nazarizadeh M, Nováková M, Loot G, Gabagambi NP, Fatemizadeh F, Osano O, Presswell B, Poulin R, Vitál Z, Scholz T, et al. 2023. Historical dispersal and host-switching formed the evolutionary history of a globally distributed multi-host parasite – the Ligula intestinalis species complex. Mol. Phylogenet. Evol. 180: 107677.

Pickrell J, Pritchard J. 2012. Inference of population splits and mixtures from genome-wide allele frequency data. Nat. Preced.: 1.

Rochette NC, Rivera-Colón AG, Catchen JM. 2019. Stacks 2: analytical methods for paired-end sequencing improve RADseq-based population genomics. Mol. Ecol. 28: 4737–4754.

Whitlock MC, Lotterhos KE. 2015. Reliable detection of loci responsible for local adaptation: inference of a null model through trimming the distribution of F_{ST}. Am. Nat. 186: S24–S36.
